# Loss of EGR-1 uncouples compensatory responses of pancreatic β cells

**DOI:** 10.7150/thno.40664

**Published:** 2020-03-04

**Authors:** Sy-Ying Leu, Li-Hua Kuo, Wen-Tsan Weng, I-Chia Lien, Ching-Chun Yang, Tai-Tzu Hsieh, Yi-Ning Cheng, Po-Hsiu Chien, Li-Chun Ho, Shun-Hua Chen, Yan-Shen Shan, Yun-Wen Chen, Pei-Jane Tsai, Junne-Ming Sung, Yau-Sheng Tsai

**Affiliations:** 1 Institute of Clinical Medicine, College of Medicine, National Cheng Kung University, Taiwan, ROC;; 2 Institute of Basic Medical Sciences, College of Medicine, National Cheng Kung University, Taiwan, ROC;; 3 Department of Physiology, College of Medicine, National Cheng Kung University, Taiwan, ROC;; 4 Department of Microbiology and Immunology, College of Medicine, National Cheng Kung University, Taiwan, ROC;; 5 Department of Pharmacology, College of Medicine, National Cheng Kung University, Taiwan, ROC;; 6 Department of Medical Laboratory Science and Biotechnology, College of Medicine, National Cheng Kung University, Taiwan, ROC;; 7 School of Medicine, College of Medicine, I-Shou University, Kaohsiung, Taiwan, ROC;; 8 Center for Clinical Medicine Research, National Cheng Kung University Hospital, Tainan, Taiwan, ROC; 9 Department of Surgery, National Cheng Kung University Hospital, Tainan, Taiwan, ROC; 10 Division of Nephrology, Department of Internal Medicine, National Cheng Kung University Hospital, Tainan, Taiwan, ROC

**Keywords:** β-cell compensation, β-cell identity, immediate genes, islet failure, stimulus-response coupling

## Abstract

**Rationale**: Subjects unable to sustain β-cell compensation develop type 2 diabetes. Early growth response-1 protein (EGR-1), implicated in the regulation of cell differentiation, proliferation, and apoptosis, is induced by diverse metabolic challenges, such as glucose or other nutrients. Therefore, we hypothesized that deficiency of EGR-1 might influence β-cell compensation in response to metabolic overload.

**Methods**: Mice deficient in EGR-1 (*Egr1*^-/-^) were used to investigate the *in vivo* roles of EGR-1 in regulation of glucose homeostasis and beta-cell compensatory responses.

**Results**: In response to a high-fat diet, *Egr1*^-/-^ mice failed to secrete sufficient insulin to clear glucose, which was associated with lower insulin content and attenuated hypertrophic response of islets. High-fat feeding caused a dramatic impairment in glucose-stimulated insulin secretion and downregulated the expression of genes encoding glucose sensing proteins. The cells co-expressing both insulin and glucagon were dramatically upregulated in islets of high-fat-fed *Egr1*^-/-^ mice. EGR-1-deficient islets failed to maintain the transcriptional network for β-cell compensatory response. In human pancreatic tissues, *EGR1* expression correlated with the expression of β-cell compensatory genes in the non-diabetic group, but not in the diabetic group.

**Conclusion**: These results suggest that EGR-1 couples the transcriptional network to compensation for the loss of β-cell function and identity. Thus, our study highlights the early stress coupler EGR-1 as a critical factor in the development of pancreatic islet failure.

## Introduction

The pathogenesis of type 2 diabetes reflects an unbalanced dynamic interaction between environmental factors, insulin-responsive tissues, and insulin-producing cells. When insulin-responsive tissues become less sensitive to insulin or circulating nutrient levels are increased, the demand for insulin grows to maintain normoglycemia. Pancreatic β cells undergo proliferation and increase output of secreted insulin. A coordinated increase in β-cell mass, insulin biosynthesis, and secretion comprises the β-cell compensatory response to increased level of nutrients (particularly glucose and fatty acid) or growth hormones, e.g., incretin 1. Individuals who fail to respond to the increased insulin demand develop hyperglycemia, a hallmark of type 2 diabetes 1. Thus, β-cell compensatory response is considered a critical determinant of whether glucose intolerance will advance to diabetes.

Reduction in β-cell number and progressive impairment of β-cell function attenuate β-cell compensatory response 1. However, Talchai *et al.* recently proposed that it is the loss of β-cell identity, rather than β-cell death, that largely accounts for the significant impairment of β-cell function during diabetes progression 2, suggesting the importance of sustaining the fully differentiated functional state of β cells. Beta-cell identity is defined by the presence of a specific gene expression signature and insulin 3. Maintenance of β-cell identity in pancreatic islets requires a dedicated transcriptional network 4. For example, PDX1 deletion in postnatal islets resulted in a loss of β cells 5, whereas β cells with NKX6.1 deletion assumed characteristics of δ cells 6. In addition, PAX4 is essential for the differentiation of β- and δ-cell lineages, whereas ARX is involved in the specification of α- and pancreatic polypeptide-cell fates 7. Moreover, other transcription factors, including NKX2.2*,* PAX6, and MAFA, have been linked to the maintenance and development of functional β cells 2, 8-10. Therefore, transcription factors involved in the pancreatic lineage development play a vital role in the mechanism that allows β cells to maintain identity and adapt to changing metabolic demands that occur throughout life 11.

It is conceivable that there is a mechanism that senses hostile environment and detects toxic effects of nutrients, switching the transcription cascade that maintains β-cell function and identity. Early growth response-1 (EGR-1), an immediate-early transcription factor, is the earliest downstream nuclear target sensitive to changes in the extracellular environment. EGR-1 is barely expressed in the basal state. Many different stimulations, including those with growth factors, hormones, cytokines, and stress inducers, promptly induced EGR-1 expression 12. The newly synthesized EGR-1 couples early extracellular stimuli to long-term responses by changing expression of the target genes. Our previous study showed that EGR-1 is rapidly and transiently induced by fatty acids, leading to the rescue of β cells from fatty acid-induced ER stress and apoptosis 13. Those results indicated that EGR-1 is a critical early sensor in pancreatic β cells that enables cellular adaptation in response to fatty acid toxicity. Thus, inhibition of this stimulus-transcription coupling machinery would impair maintenance and development of β cells, and underlie the development of diabetes in conditions of the metabolic stress.

Although EGR-1 expression is low in most tissues, it is highly enriched in pancreatic islets 14. Glucose and other secretagogues have been shown to rapidly induce EGR-1 expression in insulinoma and in primary islet cells 15, 16. Functional studies revealed that EGR-1 controls insulin biosynthesis via regulation of PDX1 expression 17-19. These studies implicate a role of EGR-1 in the transcriptional cascade that regulates β-cell identity. In addition, EGR-1 was shown to regulate β-cell proliferation 14 and rescue β cells from ER stress and apoptosis by enhancing insulin/AKT signaling in our previous study 13. Although expressing a dominant-negative mutant of EGR-1 in pancreatic β-cells has been shown to impair insulin synthesis, glucose homeostasis and islet size *in vivo* 19, the *bona fide* nature of EGR-1 in the stimulus-transcription coupling of β-cell compensatory response and identity has remained unclear. In this study, we hypothesized that the loss of EGR-1 uncouples metabolic stress from the transcriptional cascade, which is essential for augmenting β-cell compensatory response and maintenance of β-cell identity. We tested whether EGR-1 directly regulates glucose homeostasis and β-cell compensatory responses using EGR-1-deficient (*Egr1*^-/-^) mice.

## Materials and Methods

### Animals

*Egr1*^-/-^ mice, kindly provided by J. Milbrandt, Washington University in St. Louis, St. Louis, Missouri, USA 20, were maintained on a C57BL/6 genetic background. Studies were carried out using male mice and age-matched littermate wild-type (WT) mice. Mice were fed *ad libitum* either a regular chow (RC; Laboratory Rodent Diet 5001, Purina, St. Louis, MO, USA) or a high-fat diet (HF; D12492, Research Diets, New Brunswick, NJ, USA) for two to three months starting at 2 months of age. Animals were handled following procedures approved by the Institutional Animal Care and Use Committees of National Cheng Kung University.

### Glucose metabolic assays

Mice were fasted for 5 h and given an oral glucose bolus (2 g/kg body weight) or intraperitoneally injected with human regular insulin (0.4 U/kg body weight, Eli Lilly, Indianapolis, IN, USA). Blood samples were collected before and at 15, 30, 60, and 120 min after injections. Plasma glucose concentration was determined by a glucose colorimetric test (Autokit Glucose, Wako, Osaka, Japan). Insulin was measured using mouse insulin ELISA (Ultrasensitive Mouse Insulin ELISA, Mercodia, Uppsala, Sweden). The insulin resistance (IR) index was calculated as the product of the areas under glucose and insulin curves in glucose tolerance tests as previously described 21. For acute insulin secretion assay, mice were overnight fasted, and intraperitoneally injected with glucose (3 or 4 g/kg). Blood samples were collected at 0, 2, 5, 15, and 30 min after the injection.

### Morphological analysis

Serial paraffin sections with 5 μm in thickness from four different levels of pancreas (100 μm apart) were stained with H&E. The cross sectional area of islet and total tissue in four overlapping images were analyzed by ImageJ software 22. The minimum size that is considered an islet is a diameter above 50 μm. Mean islet size is calculated by dividing the total islet area by the islet number. The ratio of islet area versus total tissue area was calculated and then multiplied by the weight of the pancreas to represent the endocrine mass. Similarly, insulin-stained section was used to determine relative cross-sectional area of β cells (insulin-positive) and calculated for β-cell mass 23. Quantification of β-cell proliferation and apoptosis was done by counting the insulin-positive cells with Ki67 and caspase-3 signals, respectively. At least 500 β cells were counted for each experimental condition.

### Pancreatic islet isolation and dispersion

Pancreatic islets were isolated using a modified protocol 24. Briefly, ampulla was clamped and collagenase XI (1 mg/mL, Sigma-Aldrich, St. Louis, MO, USA) was injected into the common bile duct. The pancreata were removed and incubated, and islets were separated by sedimentation and hand-pick. To obtain isolated single islet cells, islets were dispersed with 0.1% trypsin. Isolated cells were seeded onto glass coverslips pre-coated with poly_-L-_lysine (Sigma-Aldrich, St. Louis, MO, USA) for treatment and immunofluorescence staining.

### Islet RNA analysis

All islets from individual animals were collected and extracted using the TRIzol Reagent (Invitrogen, Carlsbad, CA, USA). mRNA were analyzed with SYBR green-based real-time quantitative RT-PCR (Applied Biosystems, Foster City, CA, USA) with *Actb* as the reference gene in each reaction. Sequences of the primers used for RT-PCR assays are shown in Table S1.

### Glucose-stimulated insulin secretion

Batches of ten isolated islets were transferred to incubation vials containing pre-gassed Krebs-Ringer HEPES bicarbonate buffer in the presence of 2.8 mM glucose. After incubation for 1 h, batch triplicates were transferred to a solution containing 16.7 mM glucose for 1 h, and supernatant fractions were collected and assayed for insulin ELISA.

### Western blot analysis

Animal tissue lysates in RIPA lysis buffer were subjected to electrophoresis and transferred onto the polyvinylidene difluoride membranes (Millipore, Bedford, MA, USA). Insulin, p35, CDK5, PCNA, CDC2, pS473-AKT, AKT, pT202/204-ERK1/2, ERK1/ 2, pS217/221-MEK1/2, MEK1/2, pS21/9-GSK3α/β, GSK3α/β (Cell Signaling, Danvers, MA,USA) on the membrane were probed with respective primary antibodies and horseradish peroxidase (HRP)- conjugated secondary antibodies (Vector Laboratories, Burlingame CA, USA). Immunoreactivity was visualized by ECL plus luminal solution (GE Healthcare, Pittsburgh, PA, USA).

### Immunofluorescence staining

Paraffin-section containing pancreas tissue were deparaffinized, dehydrated, and boiled for epitope retrieval using an antigen retrieval buffer at pH = 6.0 (Opal 4-color IHC Kit, PerkinElmer, Waltham, MA). Tissue sections were blocked for 10 min and incubated with primary antibodies overnight at 4°C. Primary antibodies include: MAFA, PDX1 (Cell Signaling, Danvers, MA, USA), pancreatic polypeptide, somatostatin, NKX2.2, PAX4, PAX6, ARX, MAFA (Santa Cruz Biotechnology, Dallas, TX, USA), Ki67, PDX1 (Abcam, Cambridge, UK), glucagon and insulin (Sigma-Aldrich, St. Louis, MO, USA). The sections were incubated with Polymer HRP- conjugated secondary antibodies for 10 min at room temperature. The primary antibodies were stained with repeat procedures including antigen stripping and blocking steps. Cell nuclei was stained via adding mounting media containing fluoroshield with DAPI. The images were visualized by confocal microscopy (C1-Si, Nikon). To quantify colocalization, we used the Pearson's correlation coefficient (R_r_). The images were analyzed by using PSC Colocalization plug-in (ImageJ) 25, 26. R ranges between -1 (perfect negative correlation) to +1 (perfect positive correlation) with 0 meaning no correlation.

### Bioinformatics analysis

The PROMO database was used to identify proteins that contain potential EGR-1 binding site within endocrine lineage developmental program. Functional analysis of interacting proteins was determined using a commercial System Biology package, Ingenuity Pathways Analysis (IPA 5.0, Ingenuity Systems), following the application protocols. The results from the quantitative PCR were loaded into the IPA program to visualize the associated changes by EGR-1 deficiency or HF feeding.

### Cell culture

MIN6 cells were maintained in high-glucose DMEM supplemented with 10 % FBS. For EGR-1 overexpression, cells were transfected with plasmids expressing EGR-1 or control pCMV vector using a transfection reagent (Lipofectamine 3000, Invitrogen). For EGR-1 knockdown, EGR-1 short hairpin RNA (shEGR-1, TRCN0000301750) and control shLUC (TRCN0000072249) constructs from National RNAi Core Facility were integrated into a lentiviral delivery system as described previously 13.

### Chromatin immunoprecipitation (ChIP) assay

The procedure for ChIP was described previously 27. In brief, the interaction between protein and DNA was fixed by using 1% formaldehyde for 10 min. Cells were harvested and sonicated to fragment DNA (average size of 200-500 bp). EGR-1 antibody (#4154S; Cell signaling) was used to immunoprecipitate the EGR-1 protein and DNA complexes. Potential EGR-1 binding sites were amplified by specific primers (Table S2) after reversing cross-linking.

### EGR-1 overexpression in mice

We injected a single dose of EGR-1 plasmid (35 μg pCMV-EGR-1 plasmid/1.6 ml saline/mouse) into HF-fed WT mice by hydrodynamics-based gene delivery through the tail vein as described previously 28. Mice received the empty vector pCMV (35 μg pCMV plasmid/1.6 ml saline/mouse) as controls. The animals were euthanized at days 0, 1, 3, 7, or 10 after plasmid administration. Liver and pancreas tissues were analyzed for protein abundance. Because expression of EGR-1 was rapidly increased at day 3 after a single injection of EGR-1 plasmid, we then performed acute *in vivo* insulin secretion assay at day 3 in the HF-fed WT mice.

### Collection of human pancreas tissues

Normal pancreas specimens were obtained from 58 patients who underwent pancreatic surgery for pancreatitis or pancreatic cancer at the Department of Surgery of National Cheng Kung University Hospital from Jan. 2013 to Apr. 2014. Normal structure containing islets from pancreatic tissues was confirmed by microscopic examination. Ethics approval for human studies was obtained from the Institutional Review Board of National Cheng Kung University Hospital, and informed consent was given by each patient.

### Data analysis

Values are reported as mean ± SEM. Statistical analyses were conducted by Student's *t*-test or one-way ANOVA followed by Bonferroni multiple comparison test. Correlation was evaluated using Spearman's rank correlation coefficient. Differences were considered to be statistically significant at *P*<0.05.

## Results

### Impaired glucose homeostasis in *Egr1*^-/-^ mice after high-fat diet feeding

**Figure 1:**
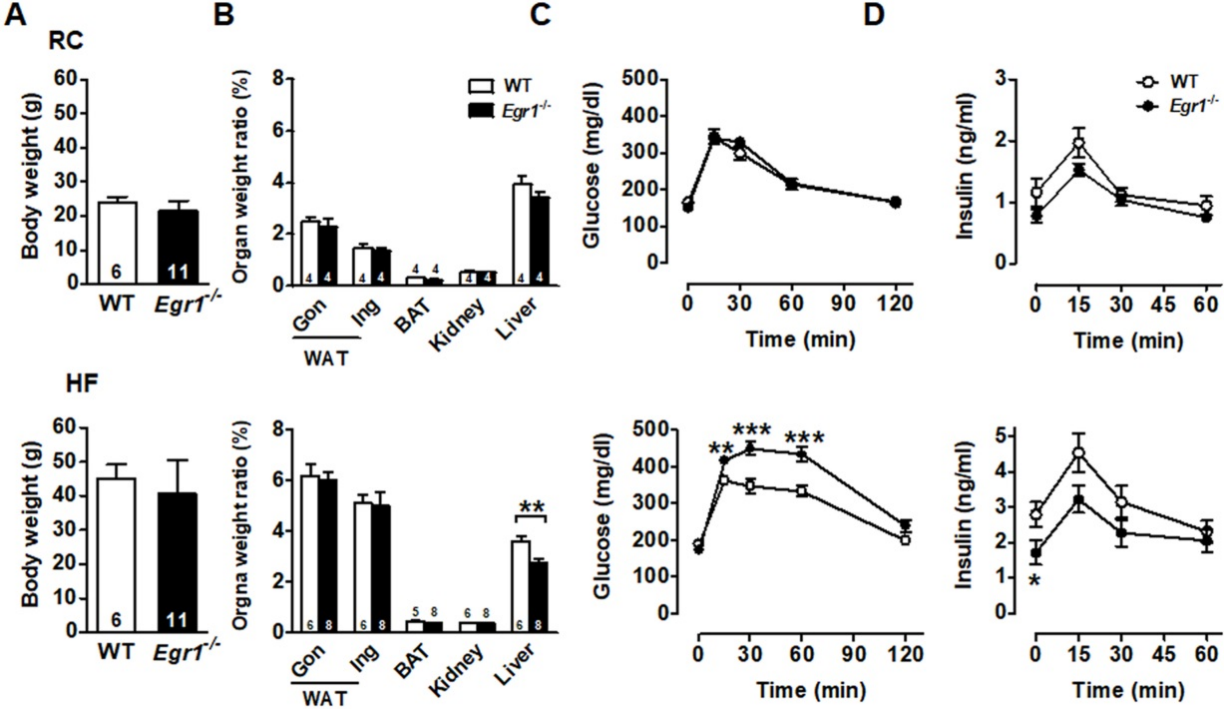
Glucose intolerance and reduced insulin levels in *Egr1*^-/-^ mice. *A*: Body weights of male mice fed RC at 2-month-old and a HF diet for 2 months starting at 2 months of age. Numbers of mice are inside bars. *B*: Organ weight ratios in 4-month-old male mice fed RC (upper panel) and a HF diet (lower panel) for 2 months. Gon, gonadal; Ing, inguinal WAT, white adipose tissue; BAT, brown adipose tissue. *C* and *D*: Plasma (*C*) glucose and (*D*) insulin levels during OGTT in 4-month-old male mice fed RC (upper panels) and a HF diet (lower panels) for 2 months. WT RC, *n*=6; *Egr1*^-/-^ RC, *n*=11; WT HF, *n*=16; *Egr1*^-/-^ HF, *n*=15. *P<0.05, **P<0.01 and ***P<0.001 compared to WT.

The body weight was similar in wild-type (WT) and *Egr1*^-/-^ mice in both basal (regular chow, RC) and enhanced metabolic demand (high-fat, HF) conditions (Figures 1*A* and S1). Except for the lower liver weight in *Egr1*^-/-^ mice, organ weights of white adipose tissue, including gonadal and inguinal fat, brown adipose tissue, and kidney did not differ between genotypes regardless of diets (Figure 1*B*). To assess the effect of EGR-1 deficiency on whole-body glucose utilization, we performed an oral glucose tolerance test. Although *Egr1*^-/-^ mice fed RC cleared glucose as efficiently as WT mice (Figure 1*C*, upper panel), we found a slight reduction in insulin levels of RC-fed* Egr1*^-/-^ mice during the oral glucose tolerance test (Figure 1*D*, upper panel). When mice were placed on the HF diet, *Egr1*^-/-^ mice could not efficiently clear glucose and had significantly lower insulin levels (lower panels of Figure 1*C* and *D*). These results suggest that *Egr1*^-/-^ mice responded normally to glucose challenge in the basal condition, but were incapable of handling glucose challenge when insulin demand was increased. Consistent with the data from previous studies 29, 30, the insulin tolerance test showed that *Egr1*^-/-^ mice were protected from diet-induced insulin resistance (Figure S2*A*). Moreover, compared to HF-fed WT mice, HF-fed *Egr1*^-/-^ mice had higher phosphorylation levels of IRS1 Tyr896 and Akt Ser473 in the liver (Figure S2*B*), and higher energy expenditure, as measured by oxygen consumption (VO_2_) and carbon dioxide production (VCO_2_) during both dark and light phases (Figure S2*C*).

### Reduced islet size and insulin content in *Egr1*^-/-^ mice after HF diet consumption

**Figure 2:**
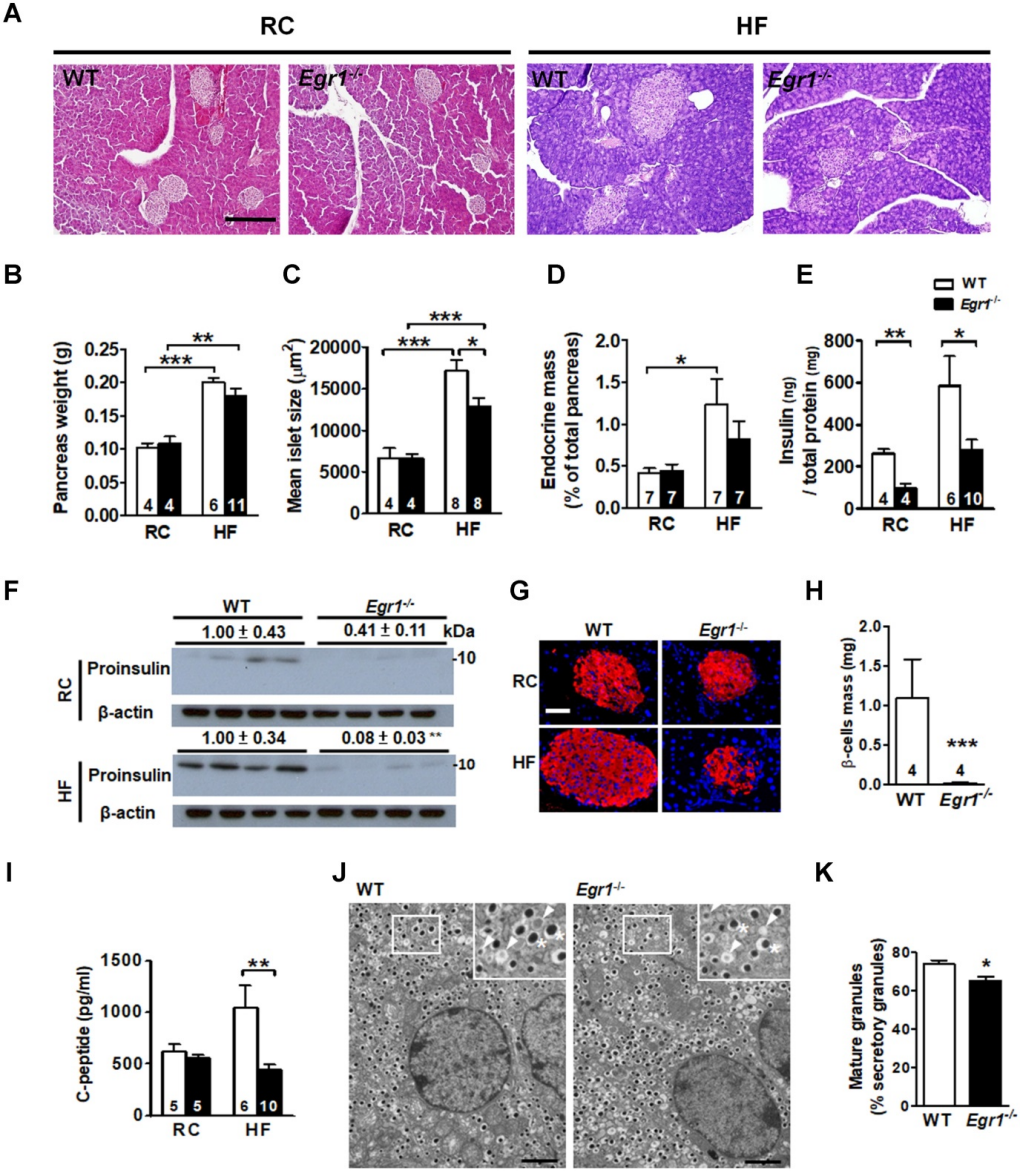
Failure to increase islet size and insulin content in response to HF feeding in *Egr1*^-/-^ mice. *A*: Morphology of pancreas from 5-month-old male mice fed RC and a HF for 3 months. Scale bar: 200 μm. *B*: Pancreas weight (g), *C*: mean islet size (μm^2^), *D*: endocrine mass (%) were measured in sections from pancreas of RC- and HF-fed mice. *E* and *F*: Insulin protein levels in the pancreas lysate determined by (*E*) ELISA or (*F*) immunoblot. The relative intensities of the bands by densitometric quantification to WT are indicated. *G*: Immunofluorescence images (Insulin, *red*; Nuclei, *blue*) in the pancreatic tissue from 5-month-old male mice fed RC and a HF diet for 3 months. (Scale bars, 50 μm). *H*: β-cell mass (mg) in sections from pancreas of 5-month-old male mice fed a HF diet for 3 months.* I*: Fasting plasma C-peptide levels from 4-month-old male mice fed RC and a HF diet for 2 months. Numbers of mice are inside bars. *J*: Representative transmission electron microscopic images of pancreatic β-cell of HF-fed WT and *Egr1*^-/-^ mice. Scale bar, 2 μm. Photomicrograph at upper right illustrates mature granules (asterisk) and immature granules (arrowhead) at a higher magnification. *K*: Percentage of mature granules. Averages of granule percentages inside bars were from 8 fields for each genotype (40 μm^2^ for each field). *P<0.05, **P<0.01, and ***P<0.001.

Given that increases in β-cell mass and insulin biosynthesis are important in islet compensatory response; we first determined the involvement of EGR-1 in the increase of β-cell mass. Pancreas weight in *Egr1*^-/-^ mice did not differ from that of WT mice regardless of diets (Figure 2*A* and *B*). Islet morphometric analysis revealed normal islet size and endocrine mass of RC-fed* Egr1*^-/-^ mice (Figure 2*C* and *D*). HF feeding doubled pancreas weight as well as tripled islet size and endocrine mass in WT mice, reflecting a compensatory islet hypertrophic response. Although *Egr1*^-/-^ mice increased pancreas weight in response to HF-feeding, the increases in the islet size and endocrine mass in *Egr1*^-/-^ mice were attenuated. Thus, attenuation of the increases of islet size and endocrine mass may explain the failure to handle glucose challenge in HF-fed *Egr1*^-/-^ mice.

We next determined insulin content in the pancreas lysate, and found that insulin protein levels were reduced regardless of diet (Figure 2*E* and *F*). Insulin intensity within islets of RC-fed *Egr1*^-/-^ mice was slightly lower than that in islets of RC-fed WT mice (Figure 2*G*). HF feeding increased islet size and insulin intensity in WT mice, but these increases was attenuated in *Egr1*^-/-^ mice (Figure 2*G*). Consistently, the levels of β-cell mass and C-peptide were significantly reduced in HF-fed *Egr1*^-/-^ mice (Figure 2*H* and *I*). We further examined insulin secretory vesicle in β cells from HF-fed WT and *Egr1*^-/-^ mice by transmission electron microscopy (Figure 2*J*). Ultrastructural analysis according to Orci *et al* 31 showed that the proportion of mature vesicles, which contain electron-dense granules, was lower in HF-fed* Egr1*^-/-^ mice (Figure 2*K*). The proportion of immature vesicles, which contain diffused gray granules, was increased in HF-fed* Egr1*^-/-^ mice. Together, these results indicate that EGR-1 deficiency impaired insulin biosynthesis and affected the formation of insulin secretory vesicles.

### Reduced glucose-stimulated insulin secretion in *Egr1*^-/-^ mice after HF diet consumption

**Figure 3:**
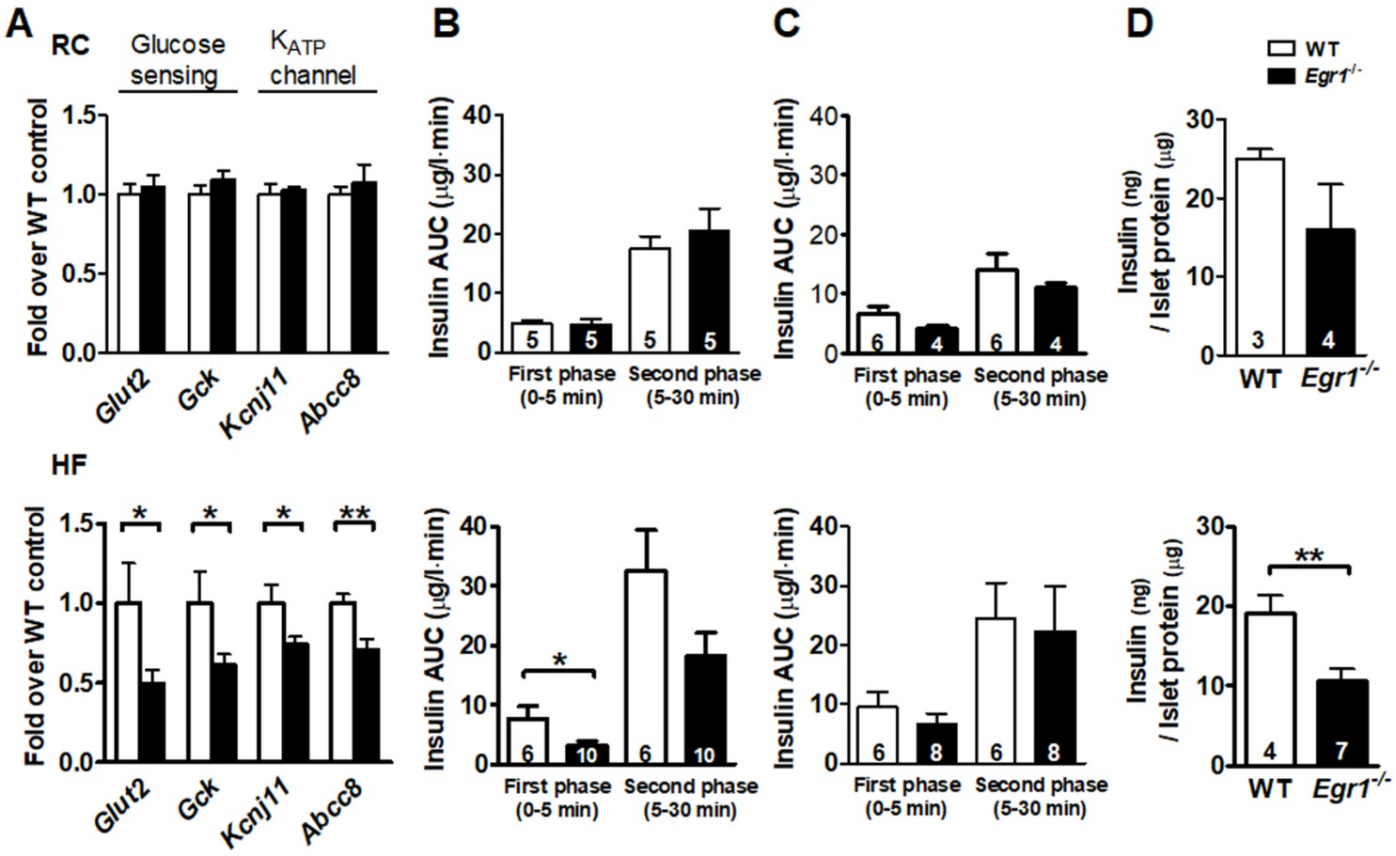
Impaired insulin secretory response to glucose challenge in* Egr1*^-/-^ mice after HF feeding. *A*: Expression of genes for glucose sensing and secretion coupling in isolated islets of 5-month-old male mice fed RC (upper panel) and a HF diet (lower panel) for 3 months. *B*: *In vivo* glucose-stimulated insulin secretion (GSIS) and *C*: arginine-stimulated insulin secretion (ASIS) in 4-month-old male mice fed RC (upper panel) or a HF diet (lower panel) for 2 months after intraperitoneal challenge of glucose (3 g/kg body weight) and arginine (0.3 g/kg body weight), respectively. *D*: *Ex vivo* GSIS in isolated pancreatic islets from male mice fed RC (upper panel) or a HF diet (lower panel). Numbers of mice are inside bars. *P<0.05 and **P<0.01.

Given that β-cell compensation also involves its responsiveness of nutrient-secretion coupling, we then tested its sensitivity to the nutrient stimuli. We first measured expression of genes involved in glucose sensing and secretion coupling from isolated islets. Expression of genes encoding proteins necessary for glucose entry, such as glucose transporter (*Glut2*) and glucokinase (*Gck*), and secretion coupling, such as ATP-sensitive potassium channels KIR6.2 (*Kcnj11*) and SUR1 (*Abcc8*), did not differ between RC-fed *Egr1*^-/-^ and WT mice (Figure 3*A*, upper panel). However, expression levels of these genes were significantly lower in HF-fed *Egr1*^-/-^ mice than in HF-fed WT mice (Figure 3*A*, lower panel). We next performed acute *in vivo* insulin secretion assay to examine the machinery of glucose sensing and insulin secretion in *Egr1*^-/-^ mice. Whereas RC-fed *Egr1*^-/-^ mice secreted normal amount of insulin in response to exogenous glucose, HF-fed *Egr1*^-/-^ mice showed attenuated glucose-stimulated insulin secretion (GSIS) (Figure 3*B*). However, insulin secretion in response to L-arginine was similar in *Egr1^-/-^* and WT mice regardless of diets (Figure 3*C*). We then isolated pancreatic islets and performed GSIS* ex vivo*. Exposure of islets to 16.7 mmol/L glucose resulted in insulin secretion into the culture medium. Islets from RC-fed *Egr1*^-/-^ mice tended to secrete less insulin, and islets from HF-fed *Egr1*^-/-^ mice showed significantly attenuated insulin secretion (Figure 3*D*). Taken together, EGR-1 deficiency, in conjunction with HF diet consumption, impaired the nutrient-secretion coupling, which was likely mediated through the downregulation of glucose-sensing machinery.

### Loss of EGR-1 attenuates the survival pathway in the islet

**Figure 4:**
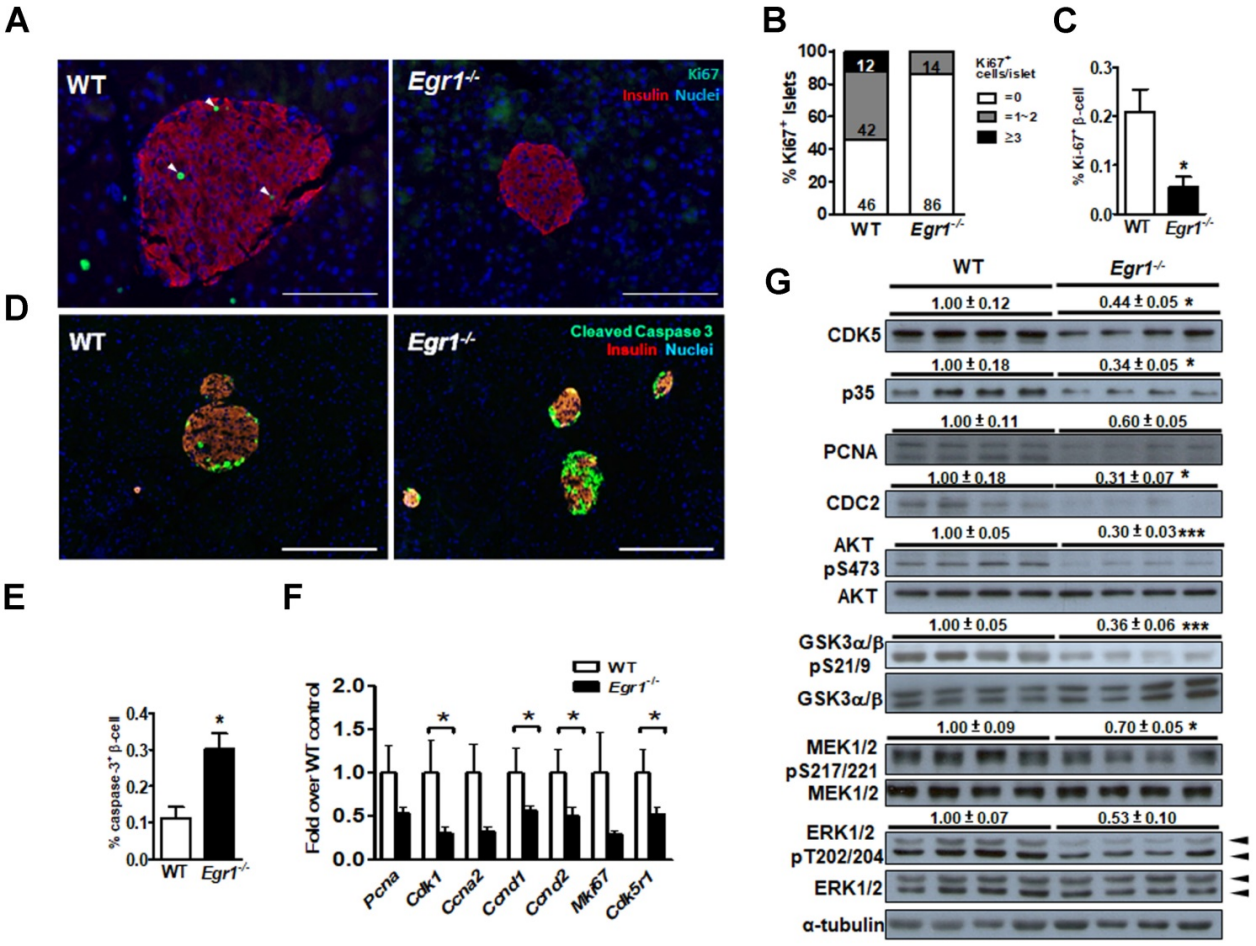
EGR-1 deficiency exhibits reduced proliferation and neogenesis in the islet upon HF feeding. *A*: Immunofluorescence staining for Ki67 (*green*) and *B*: quantification of Ki67^+^ cells in the pancreas of 5-month-old male WT (*n*=4) and *Egr1*^-/-^ (*n*=4) mice fed a HF diet for 3 months. Numbers inside bars are accumulated percentages of different categories of Ki67^+^ cell number per islet. *C*: Quantification of Ki67-positive insulin-positive cells. Scale bar: 50 μm. *D*: Immunofluorescence staining for cleaved caspase-3 (*green*) in the pancreatic islets. Images are co-stained for insulin (*red*) and nuclei (*blue*). Scale bar: 200 μm. *E*: Quantification of caspases-3-positive insulin-positive cells. *F*: mRNA levels of proliferation markers in the isolated islets of HF-fed* Egr1*^-/-^ (*n*=10) mice relative to WT (*n*=6) mice. *G*: Immunoblot analysis on proliferation-related markers and signaling molecules. The relative intensities of the bands by densitometric quantification to WT are indicated. *P<0.05 and ***P<0.001.

Because we previously found that EGR-1 deficiency enhanced palmitic acid-induced apoptosis in β cells 13, we speculated that the impairment of β-cell compensation during EGR-1 deficiency is attributed to compromised survival of islet cells. We found that Ki67-positive β cells were significantly reduced in the islets of HF-fed *Egr1*^-/-^ mice compared with their number in the islets of HF-fed WT mice (Figure 4*A*, *B*, and *C*). Moreover, HF-fed* Egr1*^-/-^ mice showed an increased cleaved caspase-3 signal in β cells (Figure 4D and E). The regulation of β-cell survival is mediated by CDK5/p35 activation and PI3K/AKT signaling pathways 32. AKT has also been proven to modulate β-cell proliferation by regulating cyclin D1 and cyclin D2 levels 33. We observed that expression levels of proliferation-related genes, including PCNA (*Pcna*), Ki67 (*Mki67*), p35 (*Cdk5r1*), CDC2 (*Cdk1*), cyclin A2 (*Ccna2*), cyclin D1 (*Ccnd1*), and cyclin D2 (*Ccnd2*), were decreased in HF-fed *Egr1*^-/-^ mice compared to those in HF-fed WT mice (Figure 4F). CDK5, p35, PCNA and CDC2 protein levels were consistently reduced in the pancreas of HF-fed *Egr1*^-/-^ mice (Figure 4*G*). Furthermore, phosphorylation of AKT Ser473, which is crucial for β-cell growth and survival 32, as well as Ser21/9 phosphorylation of its substrate GSK-3α/β, was significantly decreased in the pancreas of HF-fed *Egr1*^-/-^ mice. In addition, phosphorylation of MEK1/2 and ERK1/2, which are implicated in the regulation of expression of survival genes and prevention of apoptosis 34, was also decreased in the pancreas of HF-fed *Egr1*^-/-^ mice. Together, these results suggest that EGR-1 deficiency attenuates islet cell proliferation possibly through the downregulation of multiple signaling pathways, including CDK5/p35-mediated AKT and MEK/ERK pathways, in the HF-induced compensatory response.

### Loss of β-cell identity in the islets of HF-fed *Egr1*^-/-^ mice

**Figure 5:**
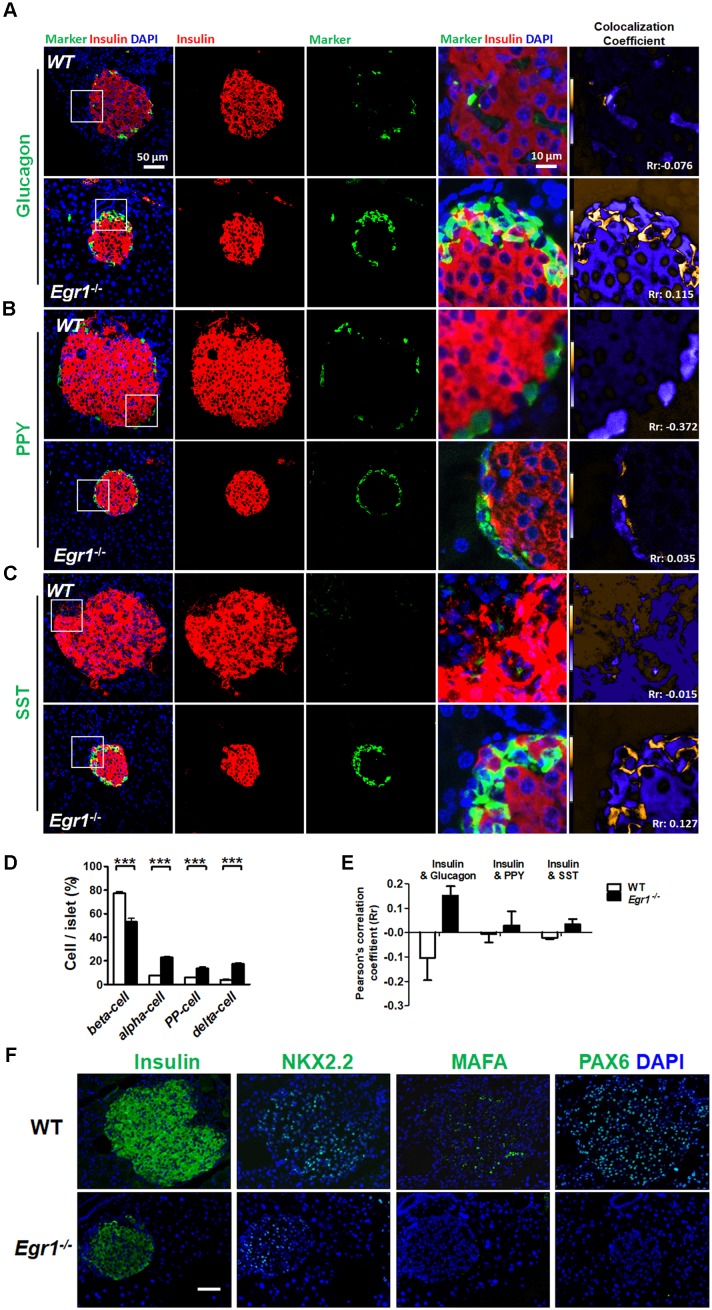
Loss of β-cell identity in the islets of HF-fed *Egr1*^-/-^ mice. Confocal images of co-staining for the markers of β-cell (insulin) in *red* with (*A*) α-cell (glucagon), (*B*) PP-cell (pancreatic polypeptide, PPY) or (*C*) δ-cell (somatostatin, SST) in *green* in the pancreas of 5-month-old male* Egr1*^-/-^ and WT mice fed a HF diet for 3 months. The enlarged images highlight the representative co-localization with 5× magnification from white squares in the overlay images. The co-localization coefficient is presented as the product of the differences from the mean (PDM) image. Yellow or blue color pixels indicate co-localization or segregation, respectively. *D*: Quantification of the markers for β-, α-, PP-, and δ-cells in the pancreas of HF-fed* Egr1*^-/-^ and WT mice. *E*: Pearson's coefficient (R_r_) of the co-localization between insulin and other hormones in the pancreas of HF-fed* Egr1*^-/-^ and WT mice. The result ranges between -1 (perfect negative correlation) to +1 (perfect positive correlation) with 0 meaning no correlation. *F*: Immunofluorescence staining of the transcription factors critical for β-cell identity. Images are co-stained for nuclei (*blue*). ***P<0.001.

**Figure 6:**
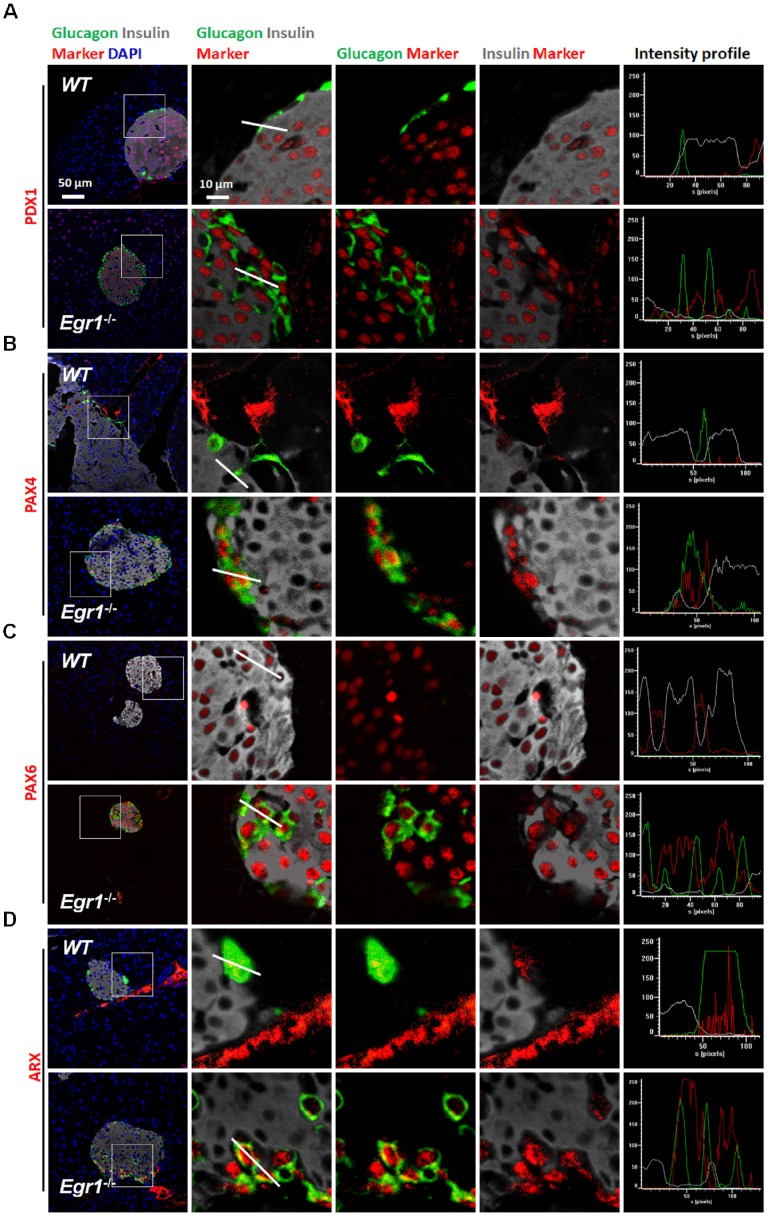
Glucagon-positive α cells expressed typical β-cell transcription factors in the islets of HF-fed *Egr1*^-/-^ mice. Confocal images of co-staining for the markers of β-cell (insulin; *white*) and α-cell (glucagon; *green*) with (*A*) PDX1, (*B*) PAX4, (*C*) PAX6, and (*D*) ARX (*red*) in the pancreas of HF-fed* Egr1*^-/-^ and WT mice. The enlarged images highlight the representative co-localization with 5× magnification from white squares in the overlay images. The fluorescence intensity profile from *green*, *red*, and *white* channels is shown.

During prolonged metabolic stress, β cells lose expression of functional markers and convert to other endocrine cell types 2. We next examined whether EGR-1 deficiency altered morphology and composition of the pancreatic islet. In contrast to the decreased percentage of insulin-positive β cells, we found increased percentages of glucagon-positive α cells, pancreatic polypeptide-positive PP cells, and somatostatin-positive δ cells in the islets of HF-fed *Egr1*^-/-^ mice (Figure 5*A*,* B*, *C*, and *D*). The decreased percentage of β cells and increased percentages of α, PP, and δ cells in the islets were observed in RC-fed* Egr1*^-/-^ mice (Figure S3), and even in the neonatal* Egr1*^-/-^ mice (Figure S4). However, we did not detect Ki67-positive PP- and δ-cells (Figure S5). Interestingly, the cells co-expressing insulin and glucagon, which are rarely found in the islets of HF-fed WT mice, were dramatically upregulated in the islets of HF-fed *Egr1*^-/-^ mice (Figure 5*A*). In addition, the islets of *Egr1*^-/-^ mice also contained other bi-hormonal cells, including those co-expressing insulin and pancreatic polypeptide as well as those co-expressing insulin and somatostatin (Figure 5*B* and *C*). Analysis of the correlation of colocalizing hormones, reflected by the Pearson's correlation coefficient, showed that the colocalizations of insulin/ glucagon, insulin/pancreatic polypeptide, and insulin/somatostatin were increased in the islets of *Egr1*^-/-^ mice (Figure 5*E*). Further characterization of the islets of *Egr1*^-/-^ mice revealed that transcription factors maintaining β-cell identity, including NKX2.2, MAFA, and PAX6, were downregulated (Figure 5*F*). Interestingly, we noticed that glucagon-labeled α cells expressed typical β-cell transcription markers, such as PDX1, PAX4, and PAX6, in the islets of HF-fed *Egr1*^-/-^ mice (Figure 6*A*, *B*, and *C*). However, expression of α-cell transcription marker ARX was limited to glucagon-labeled α-cells in the islets of HF-fed *Egr1*^-/-^ mice (Figure 6*D*). These results suggest that EGR-1 deficiency results in the loss of clear identification between α and β cells during enhanced metabolic demand.

### Decreased transcriptional network in the islets of HF-fed *Egr1*^-/-^ mice

**Figure 7:**
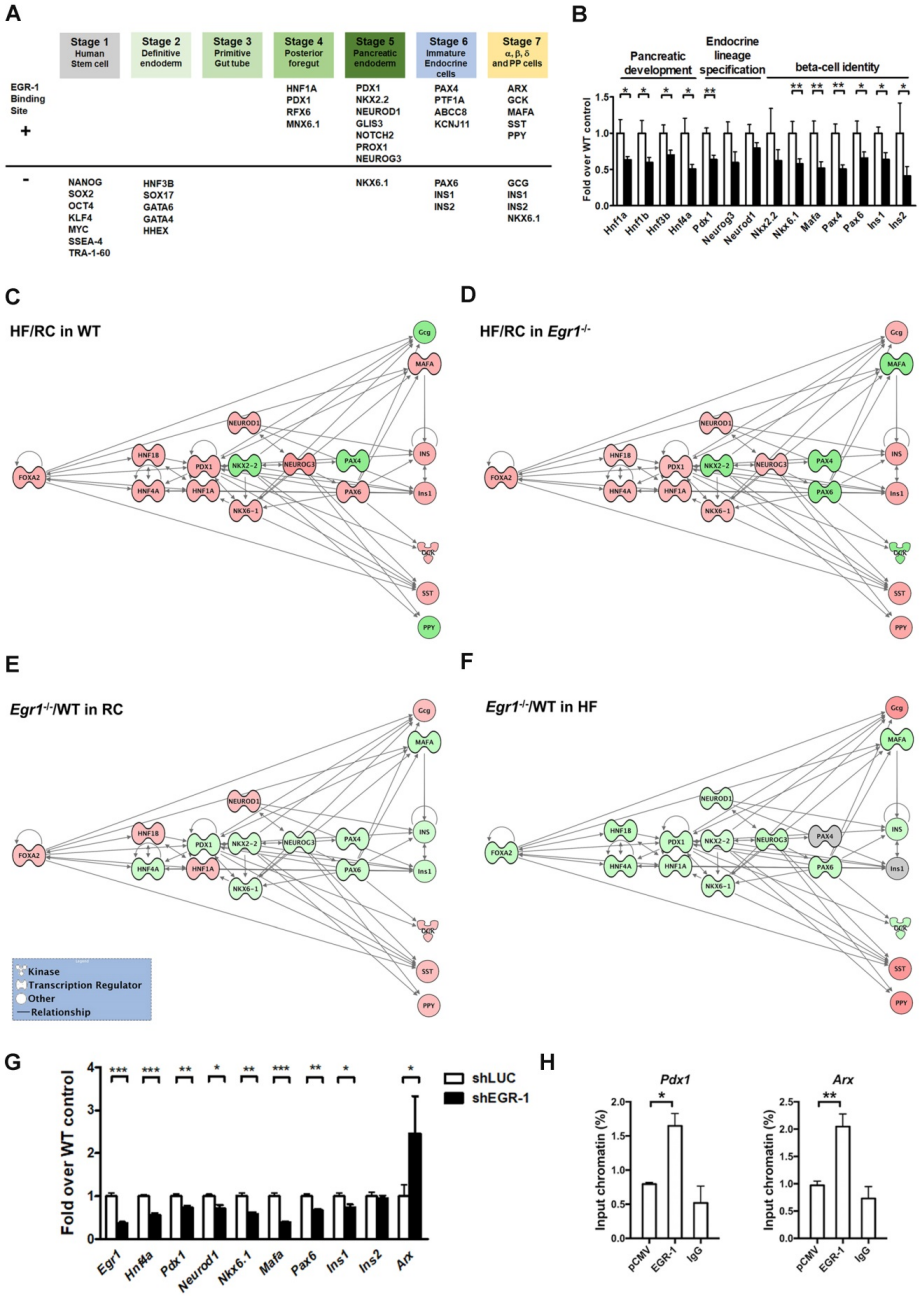
Decreased transcriptional network in the islets of HF-fed *Egr1*^-/-^ mice. *A*: Bioinformatics analysis in identification of transcriptional factors and endocrine molecules that contain potential EGR-1 binding site. Sequences for proteins involved in the development program of islet lineages were analyzed for potential EGR-1 binding site using the PROMO transcription factor binding site database. *B*: Expression of genes for pancreatic development, endocrine lineage specification and β-cell identity in the isolated islets of HF-fed* Egr1*^-/-^ (*n*=10) relative to WT (*n*=6) mice. *P<0.05 and **P<0.01 compared to WT mice. Pathway analysis of the response of HF feeding on the islets of (*C*) WT and (*D*) *Egr1*^-/-^ mice using Ingenuity Pathways Analysis (IPA) software. Pathway analysis of the effect of EGR-1 deficiency on the transcription network in (*E*) RC and (*F*) HF diet feeding. Color shading corresponds to the type of dysregulation: red for up-regulated and green for down-regulated genes according to the results of quantitative PCR. The shape of the node indicates the major function of the protein: transcription regulators (dumbbell); kinases (three arrows); and others (circle). *G*: Expression of genes in EGR-1 knockdowned (*n*=5) relative to control (*n*=5) MIN6 cells. *H*: ChIP assay in EGR-1 overexpressed and control (pCMV) MIN6 cells. Sequences containing the potential EGR-1 binding sites in *Pdx1* and *Arx* were amplified by real-time PCR. *n*=3 in each group. *P<0.05, **P<0.01, and ***P<0.001.

Given that EGR-1 deficiency attenuated several key components of β-cell compensatory response, we hypothesized that EGR-1 sets an early checkpoint for the compensatory network. To test this hypothesis, we first performed the bioinformatics analysis to screen for the transcriptional factors and endocrine molecules that contain EGR-1 binding site. The prediction results showed that 20 (out of 38) proteins within the developmental program of islet lineages 35 contain an EGR-1 binding site (Figure 7*A*). Most of the transcriptional factors carrying EGR-1 binding site are involved in the development of posterior foregut, pancreatic endoderm, and endocrine cell maturation. We next determined expression levels of genes in pancreatic islets by using quantitative PCR. Expression levels of genes encoding members of the hepatocyte nuclear factor family (HNF1A, HNF1B, HNF3B, and HNF4A), which are required for proper pancreatic development 11, 36, were significantly downregulated in the islets of HF-fed *Egr1*^-/-^ mice (Figure 7*B*). The genes encoding endocrine lineage-specified transcription factors, such as PDX1, NEUROG3, and NEUROD1 11, were also downregulated in the islets of HF-fed* Egr1*^-/-^ mice. Furthermore, expression levels of genes encoding transcription factors that maintain β-cell identity, such as NKX2.2, NKX6.1, MAFA, PAX4, and PAX6 37, 38, as well as insulins, INS1 and INS2, were downregulated in islets of HF-fed* Egr1*^-/-^ mice.

Finally, to gain further insight into the putative pathways associated with EGR-1 in the compensatory network, we performed the Ingenuity Pathways Analysis (IPA). We first examined the response of high-fat feeding on the islets of WT and *Egr1*^-/-^ mice. High-fat feeding up-regulated (*red*) several transcription factors, including PDX1, NKX6.1, NEUROD1, NEUROG3, PAX6, and MAFA, in the islets of WT mice (Figure 7*C*). Although high-fat feeding also up-regulated PDX1, NKX6.1, NEUROD1, and NEUROG3 in the islets of *Egr1*^-/-^ mice, it did not up-regulate PAX6 and MAFA (Figure 7*D*). Therefore, high-fat feeding could normally bring up several transcription factors, but the deficiency of EGR-1 causes a different response in upregulation of these transcription factors. We further examined the effect of EGR-1 deficiency on the transcription network in both regular and HF diet feeding. In regular diet, EGR-1 deficiency down-regulated (*green*) transcription factors, including HNF4A, PDX1, NKX2.2, NKX6.1, NEUROG3, PAX4, PAX6, and MAFA (Figure 7*E*). EGR-1 knockout combined with HF diet leads to further down-regulation in transcription factors FOXA2, HNF1B, HNF1A, and NEUROD1 (Figure 7*F*). Thus, EGR-1 deficiency alone has an impact on transcription network, and EGR-1 knockout combined with HF diet leads to a synergistic effect.

To solidify the effect of EGR-1 knockout in β-cells, we conducted a series of experiments in MIN6 mouse pancreatic β cells. Firstly, knockdown of EGR-1 by short hairpin RNA (shRNA) significantly decreased HNF4A, PDX1, NEUROD1, NKX6.1, MAFA, and PAX6, whereas it significantly increased ARX (Figure 7*G*). While EGR-1 knockdown modestly decreased INS1, it did not affect expression of INS2. Secondly, we further demonstrated the binding of EGR-1 to the genomic sequence of these potential target genes. Chromatin immunoprecipitation (ChIP)-PCR in MIN6 cells showed that EGR-1 bound to the promoter region of *Pdx1* and *Arx* (Figure 7*H* and Figure S6). These results suggest that EGR-1 affects β-cell function in a cell-intrinsic mechanism. Finally, we overexpressed EGR-1 in the HF-fed WT mice by hydrodynamics-based gene delivery. Expression of EGR-1 was rapidly increased at day 3 after a single injection of EGR-1 plasmid (Figure S7*A*). We then performed acute *in vivo* insulin secretion assay at day 3 in the HF-fed WT mice, and found that EGR-1 overexpression increased the second phase of GSIS (Figure S7*B*). Thus, EGR-1 plays a key role in regulation of the production of insulin and glucose homeostasis in HF-fed mice.

### Positive correlation between expression levels of EGR-1 and β-cell compensatory genes in human pancreatic tissues

**Figure 8:**
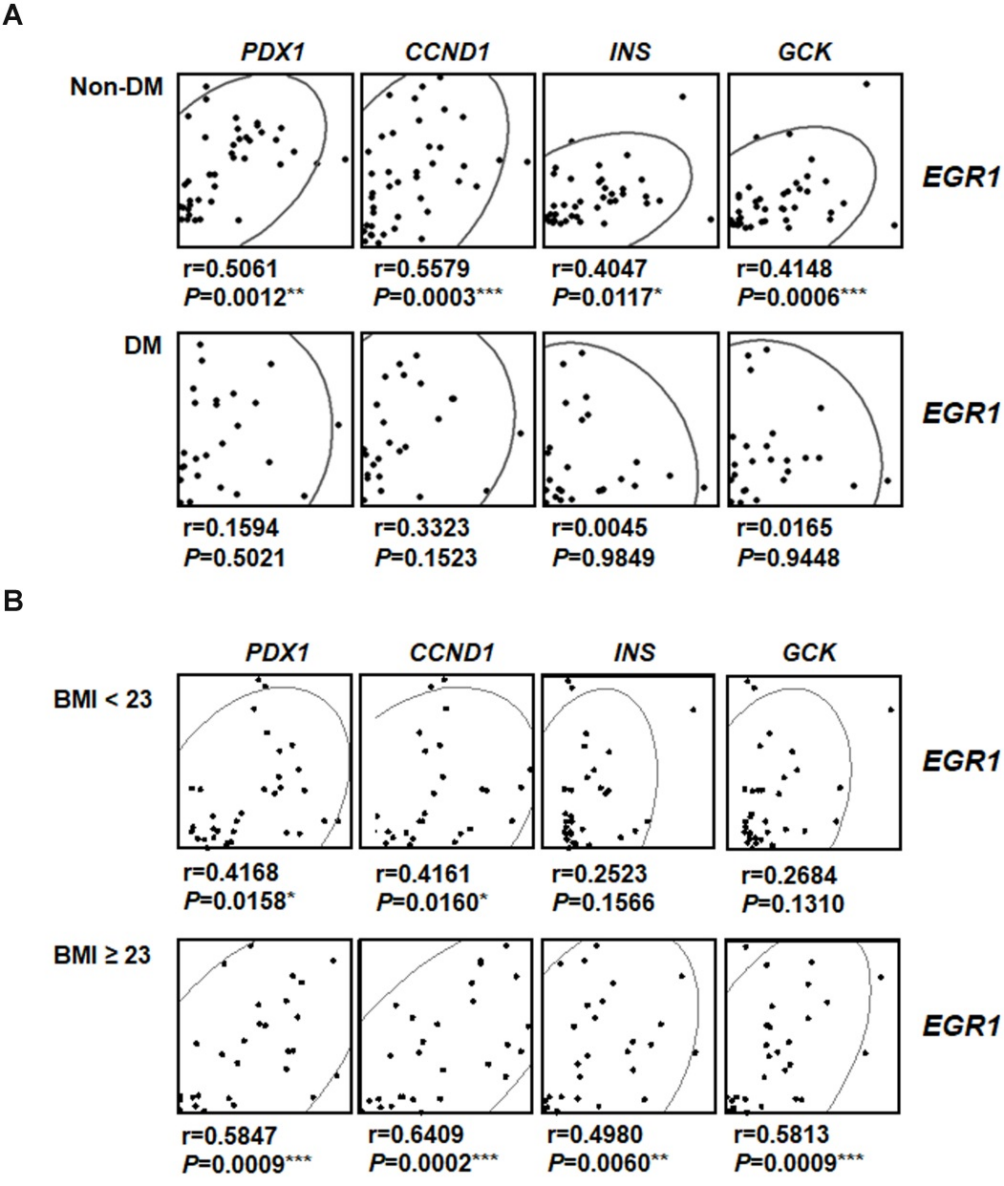
The correlation between EGR-1 expression and compensatory genes in human pancreatic tissues. Scatter plot illustrating the Spearman's correlation of normalized reads per patient between *EGR1* and *PDX1*, as well as compensatory genes, such as *CCND1* (cyclin D1), *INS* (insulin), and *GCK* (glucokinase), in (*A*) non-diabetic (upper panels) and diabetic (lower panels) subjects; and (*B*) normal weight (BMI < 23, upper panels) and overweight/obese (BMI ≥ 23, lower panels) subjects. Spearman's rank correlation coefficients *r* and *P* value are provided in each plot. *P<0.05, **P<0.01, and ***P<0.001.

To investigate the involvement of EGR-1 in human pancreatic β-cell compensatory responses, we collected 58 human pancreas samples and divided them into type 2 diabetic and non-diabetic groups (Figure 8*A*). Interestingly, we found that *EGR1* expression correlated with the expression of its downstream gene *PDX1* and β-cell compensatory genes, including *CCND1* (cyclin D1 for β-cell proliferation), *INS* (insulin), and *GCK* (glucokinase for nutrition-secretion coupling) in the non-diabetic group (Figure 8*A*, upper panels). However, these correlations were not found in the diabetic group (Figure 8*A*, lower panels). These results suggest a role of EGR-1 in the regulation of β-cell compensation, particularly in non-diabetic subjects. Moreover, we further divided our study population into 2 groups (normal weight, BMI < 23 vs. overweight/obese, BMI ≧ 23) using the Asian criteria of the World Health Organization. Although *EGR1* expression correlated with the expression of *PDX1*, *CCND1*, *INS*, and *GCK* in both groups, the correlation tends to be more significant with higher r-value and smaller p-value in the overweight/obese group than in normal weight group (Figure 8*B*).

## Discussion

This study was designed to examine β-cell compensatory response under metabolic stress and to test the hypothesis that EGR-1 plays a significant role in this stimulus-transcription coupling. Dissecting the regulation of this immediate-early gene in adaptation to metabolic stress is crucial for our understanding of type 2 diabetes development. We found that *Egr1^-/-^* mice fed a chow diet displayed normal glucose tolerance and GSIS. Upon metabolic stress, EGR-1 deficiency impaired β-cell compensatory responses, as was evidenced by impairments in insulin biosynthesis, β-cell mass expansion, and nutrient-secretion coupling. Furthermore, EGR-1 deficiency also downregulated pro-survival pathways and elicited a population of glucagon^+^ cells carrying β-cell characteristics. These defects were linked to the reductions in a number of transcription factors and signaling cascades essential to maintain the compensatory network and β-cell identity. Thus, our study identifies EGR-1 as a regulator of the transcriptional network that compensates for the loss of β-cell mass and function.

EGR-1 has been revealed as an early sensor and regulator in both physiological and pathological conditions 12. In the absence of external stimuli, EGR-1 expression is scarce. Upon stimulation by extracellular stimuli, the biosynthesis of EGR-1 is rapidly induced. EGR-1 couples extracellular stimulations to long-term responses by changing expression patterns of its target genes. In addition to sensing the hostile environment, EGR-1 has also been shown to be a glucose-responsive gene in β cells. The induction of EGR-1 expression by glucose appears to correlate with insulin secretion 39. EGR-1 expression is implicated in proliferation and transactivation of insulin gene in β cells 40. Moreover, the attenuation of EGR-1 expression likely contributed to β-cell dysfunction in diabetic rats 14. These observations are consistent with our findings that HF-fed *Egr1^-/-^* mice had reduced islet size, insulin content, and GSIS, as well as impaired glucose homeostasis. These data suggest that EGR-1 plays important roles in controlling proliferation, insulin biosynthesis, and glucose sensing of pancreatic β cells in HF-fed mice. To test whether EGR-1 serves as a candidate for clinical application, we overexpressed EGR-1 in the HF-fed mice by hydrodynamics-based gene delivery. Our results showed that of EGR-1 overexpression in the HF-fed mice increased the second phase of GSIS. Thus, the induction of the early sensor EGR-1 may be a therapeutic strategy for targeting pancreatic islet failure.

The specialized features of β cells are determined by the expression of a subset of genes controlled by several transcription factors. Many members of the hepatocyte nuclear factor (HNF) family, such as HNF1A, HNF1B, HNF3A, HNF3B, and HNF4A, are important in regulating early development of pancreas 11, 36. Although some HNFs are not predicted to contain an EGR-1 binding site, EGR-1 deficiency caused a general reduction in the levels of these HNFs. Cell lineage is determined by feed-forward and autocrine control of transcription factor gene expression 41. It is likely that EGR-1 deficiency affects expression of HNFs through the feed-forward mechanism. After pancreas formation, expression of NEUROG3 and NEUROD1 specifies endocrine precursor cell formation. At the later stage, another set of transcriptional factors, including NKX2.2, NKX6.1, MAFA, and PDX1, drive islet cell differentiation. These transcription factors are critical for the expression of genes in a mature β cell that enable its specialized function, i.e. the expression, processing, storage, and regulated secretion of insulin. These genes generally harbor binding sites for PDX1, NEUROD1, MAFA, but we also found that most of them contain EGR-1 binding sites (Figure 7A). Moreover, EGR-1 deficiency caused a general reduction in expression levels of these transcriptional factors when mice were placed in the condition of metabolic overload. These transcriptional factors are crucial for the maintenance of β-cell phenotype, and EGR-1 can be induced under special circumstances to coordinate expression of these transcriptional factors, allowing β cells to respond to stressful conditions.

Many studies have identified external stimuli and intrinsic transcriptional networks that are crucial for the generation of functional insulin-producing β cells *in vitro* and *in vivo* 42, 43. Several transcriptional factors that are essential for pancreatic development during embryogenesis also regulate the maintenance of β-cell function in the adulthood 44. This implies that transcriptional networks that are necessary for the initial specification of β cells may be re-expressed in adult β cells to maintain their identity and function in a hostile environment. However, the role of EGR-1 in the regulation of β-cell identity has not been addressed. In this study, increased numbers of glucagon^+^, pancreatic polypeptide^+^, and somatostatin^+^ cells were found in the islets of *Egr1^-/-^* mice. However, we did not detect Ki67-positive PP- and δ-cells, suggesting that these increases are not likely caused by increased proliferation of PP- and δ-cells. Thus, increased number of other endocrine cell types in the islets of *Egr1^-/-^* mice might result from the conversion of β cells. Another possibility is that the decrease of β cells in *Egr1^-/-^* mice may activate some other endocrine cell types to acquire β-like cell characteristics. Nevertheless, the origin of these non β cells with β-cell characteristics will await advanced lineage-tracing experiments. These findings suggest that EGR-1 is a critical factor that turns on transcriptional networks for the maintenance of β-cell identity. This mechanism may be important during the development of pancreatic islet failure.

A previous study by Müller* et al.* showed reduced insulin biosynthesis and islet size in transgenic mice expressing a dominant-negative mutant of EGR-1 in pancreatic β-cells 19. The reported model is based on a transgenic system impairing the biological functions of the entire EGR family. When keeping these mice with a normal diet, they already showed the evidence of reduced β-cell function. In contrast, a similar phenotype occurs in the *Egr1^-/-^* mice with a high-fat diet. We then evaluated the levels of EGR-2 and EGR-3 in the islets of *Egr1*^-/-^ mice. Our results showed that mRNA levels of EGR-2 and EGR-3 tended to be higher in *Egr1*^-/-^ mice, but their levels were extremely low in the islets (*Egr2*/*Egr1*= 0.0045 and *Egr3*/*Egr1*= 0.0002; Figure S8), suggesting a relatively minor impact of EGR-2 and EGR-3 in the islets. Nevertheless, compensation of EGR-1 loss by homologous proteins of the EGR family cannot be excluded. However, we found the discordance between two models. For instance, the expression of glucagon and somatostatin and the proliferation of β-cells did not differ between their mutant and control mice, which are likely due to low proliferation and turnover in the basal chow-fed condition. In contrast, our study applied *Egr1*^-/-^ mice, which specifically address the physiological consequence of EGR-1 deficiency during metabolic stress. In addition, the natural high-caloric diet feeding course allows us to unravel the possible transcription network and signaling cascade affected by EGR-1 deficiency under metabolic stress. Particularly, we found that WT mice fed with a HF diet still retained β-cell identity. However, *Egr1*^-/-^ mice fed with a HF diet lost this clear identification between α and β cells. Therefore, EGR-1 deficiency results in a loss of cell identity between α and β cells. Understanding how a β cell maintains its identity through EGR-1-mediated transcription network would help find a therapeutic strategy for targeting pancreatic islet failure.

It has been reported that EGR-1 could affect the insulin sensitivity and metabolism in adipose tissue, liver, and skeletal muscle. For example, Yu *et al.* showed that EGR-1 decreases adipocyte insulin sensitivity by blocking PI3K/AKT and augmenting ERK/MAPK signaling 29. Inhibition of EGR-1 activity by injecting an adenovirus carrying dominant-negative EGR-1 into epididymal fat of hyperphagic *db/db* mice improved their sensitivity to systemic insulin. Chronic exposure to insulin causes persistent ERK/MAPK activity in adipocytes and enhances insulin resistance in an EGR-1-dependent manner 45. Moreover, deletion of EGR-1 in the liver and skeletal muscle improves systemic insulin sensitivity 46, 47. Consistently, our data and published results of other groups showed that *Egr1*^-/-^ mice were protected from diet-induced insulin resistance 30. Because the assessment of insulin secretion and β-cell function could be affected by the peripheral insulin sensitivity, the role of EGR-1 in regulating β-cell mass and function should be carefully interpreted and would require either a β-cell specific EGR-1 knockout or the transplantation of wild-type β-cells into the whole body knockout model. Nevertheless, we believe that providing the information regarding how a β cell maintains its identity through EGR-1-mediated transcription network to the field is a timely issue.

Although these results suggest that EGR-1 knockout affects β-cell function, it is not clear whether this occurs through a cell-intrinsic or cell-extrinsic mechanism. It should be noted that because we employed a whole-body knockout system, it is possible that the role of EGR-1 in pancreatic β cells was masked by metabolic adaptation to EGR-1 deficiency in other relevant tissues. Therefore, the decreased expression of potential EGR-1 targets in islets does not rule out the possibility that EGR-1 deficiency in other cell types produces cell-extrinsic effects on β-cell expression of these genes. To strengthen a cell-intrinsic role of EGR-1 in β-cells, we conducted a series of experiments. Firstly, the decreased percentage of β cells and increased percentages of α, PP, and δ cells in the islets were observed in RC-fed* Egr1*^-/-^ mice, and even in the neonatal* Egr1*^-/-^ mice. These results suggest that the effect of EGR-1 knockout in β-cells is observed when the influence by peripheral tissues is relatively minimal. Secondly, EGR-1 knockdown decreased expression of a number of β-cell transcriptional factors, whereas it increased α-cell transcriptional factor in *in vitro* cell model. Thirdly, we further demonstrated the binding of EGR-1 to the promoter region of potential target genes* Pdx1* and *Arx* in MIN6 mouse pancreatic β cells. In summary, these results suggest that EGR-1 affects β-cell function in a cell-intrinsic mechanism.

To examine the clinical significance of EGR-1 in β-cell compensatory response, we collected normal pancreatic tissues from 58 human subjects and divided them into diabetic and non-diabetic groups. It has been suggested that β-cell failure occurs after an initial compensatory state of β-cell expansion in type 2 diabetes. We then hypothesized that the compensatory response would remain intact in non-diabetic subjects, but would be impaired in diabetic subjects. Indeed, we found that EGR-1 expression positively correlated with that of cyclin D1, insulin, glucokinase in non-diabetic subjects that contained a spectrum of normal and pre-diabetic conditions. However, these correlations were absent in diabetic subjects. These results suggest that EGR-1 is able to execute its role in governing the transcriptional network only when the compensatory machinery is intact.

Interestingly, there seems to exist a discordance between the effects of EGR-1 on β cells in mouse versus human islets. In mice, EGR-1 seems to have an effect on β-cell transcription only after a metabolic challenge (HF-diet). However, in humans, the effect of EGR-1 is observed in unchallenged healthy individuals and disappears in diabetic subjects. This discordance may stem from the difference in the experimental design and a physiological difference between mice and humans. For instance, in humans, we mainly focused on the correlations between EGR-1 and compensatory genes. In mice, we primarily tested the effect of EGR-1 loss on β-cell function and compensatory machinery. We suggest that the regulation of compensatory machinery by EGR-1 may take place in individuals with preserved β-cell function, but dissipate in those with impaired β-cell function. Because the HF-diet feeding in mice is more correlated with obesity, we then divided our subjects into normal weight and overweight/obese groups. The correlations of *EGR1* with *PDX1*, *CCND1*, *INS*, and *GCK* are stronger in the overweight/obese group than in normal weight group (Figure 8*B*). These results suggest that, in humans, EGR-1 tends to have a stronger effect on β-cell transcription after a metabolic challenge.

In summary, our study demonstrated that the loss of EGR-1 uncouples metabolic stress from the transcriptional cascade essential for β-cell compensatory response and identity. Thus, the early mediator EGR-1 is crucial for long-term adaptive effects during the compensatory response in β cells. We also identified EGR-1 as an important transcription factor that promotes β-cell identity, maintains β-cell characteristics, and represses non-β-cell programs. These effects are likely mediated through a series of transcriptional factors maintaining β-cell identity during metabolic stress. Understanding the molecular mechanism by which EGR-1 allows β cells retain the stable identity will be important for deriving fully differentiated, functional β cells to meet the metabolic demand. Furthermore, our study underscores the early stress coupler EGR-1 as a critical factor in the pathogenesis of pancreatic islet failure.

## Supplementary Material

Supplementary figures and tables.

## Abbreviations

EGR-1: early growth response-1
HF: high-fat
WT: wild-type
RC: regular chow
OGTT: oral glucose tolerance test
GSIS: glucose-stimulated insulin secretion
ASIS: arginine-stimulated insulin secretion
PI3K: phosphoinositide 3-kinases
AKT: serine/ threonine-specific protein kinase
MEK: mitogen- activated protein kinase kinase
ERK: extracellular signal-regulated kinases
*INS*: insulin
*GCK*: glucokinase
HNF: hepatocyte nuclear factor
IR: insulin resistance
