# Supplementary material for: Loss of EGR-1 uncouples compensatory responses of pancreatic β cells
Source: Theranostics. 2020 Mar 4;10(9):4233–49. doi: 10.7150/thno.40664 (PMC7086362; doi:10.7150/thno.40664)
Supplement: Supplementary file 1 — Supplementary figures and tables. [file thnov10p4233s1.pdf]

Figure S1

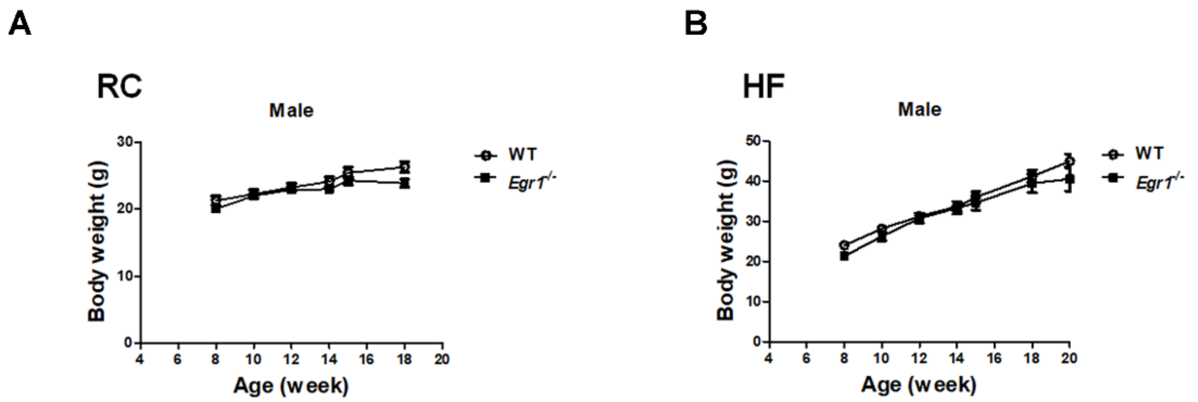

Figure S1. The body weights of 2-month-old male mice fed (A) RC and (B) a HF diet for 3 months. WT RC, *n*=3; *Egr1*$^{-/-}$ RC, *n*=4; WT HF, *n*=6; *Egr1*$^{-/-}$ HF, *n*=11.

Figure S2

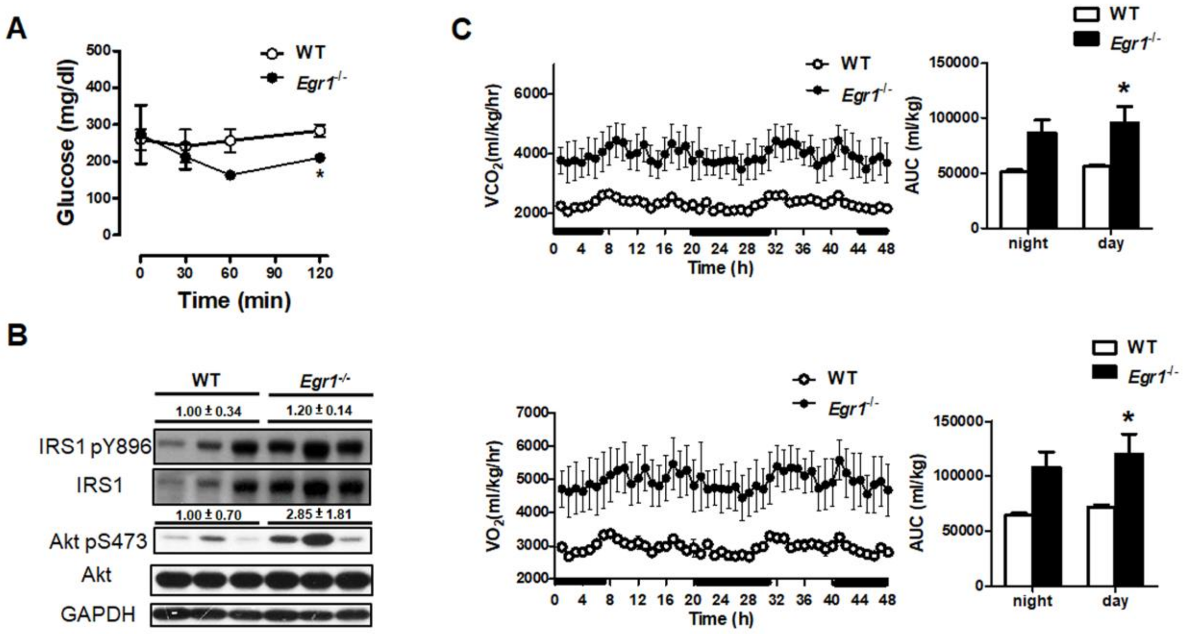

Figure S2. *Egr1*$^{-/-}$ mice are protected from diet-induced insulin resistance. *A*: Plasma glucose levels during insulin tolerance test in 5-month-old male mice fed a HF diet for 3 months. WT, n=5; *Egr1*$^{-/-}$, *n*=3. *P<0.05 compared to WT. *B*: Immunoblot analyses on phosphorylation at Tyr896 of IRS1 and Ser473 of Akt in the liver of 5-month-old male mice fed a HF diet for 3 months. Each band represents a tissue extract from a single mouse. The intensities of the bands, quantified densitometrically relative to WT, are shown. *P<0.05, **P<0.01 and ***P<0.001 for *Egr1*$^{-/-}$ mice compared with WT mice. *C*: Oxygen consumption (VO_2; upper panels) and carbon dioxide production (VCO_2; lower panels) and their mean AUC of HF-fed male mice in indirect calorimetry over 48 h (n=3 in each group). Bold bars on the x-axes represent the dark phases.

Figure S3

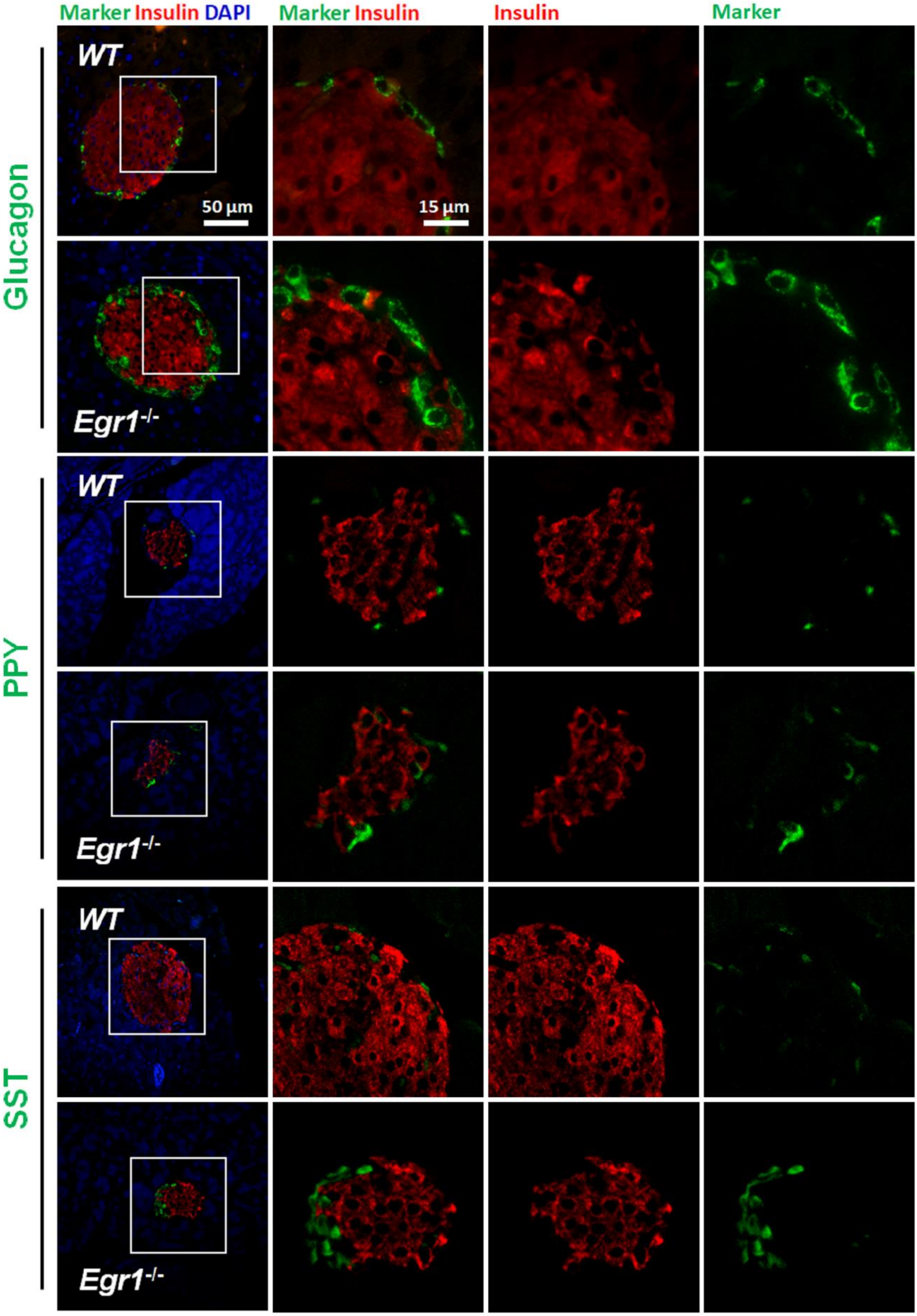
Marker Insulin DAPI
Marker Insulin
Insulin
Marker
Glucagon
PPY
SST
WT
Egr1-/-
50 µm
15 µm

Figure S3. Loss of β-cell identity in the islets of RC-fed *Egr1*$^{-/-}$ mice. Confocal images of co-staining for the markers of β-cell (insulin) in *red* with α-cell (glucagon), PP-cell (pancreatic polypeptide, PPY), or δ-cell (somatostatin, SST) in *green* in the pancreas of 5-month-old RC-fed mice. The enlarged images highlight the representative co-localization with 3× magnification from white squares in the overlay images.

Figure S4

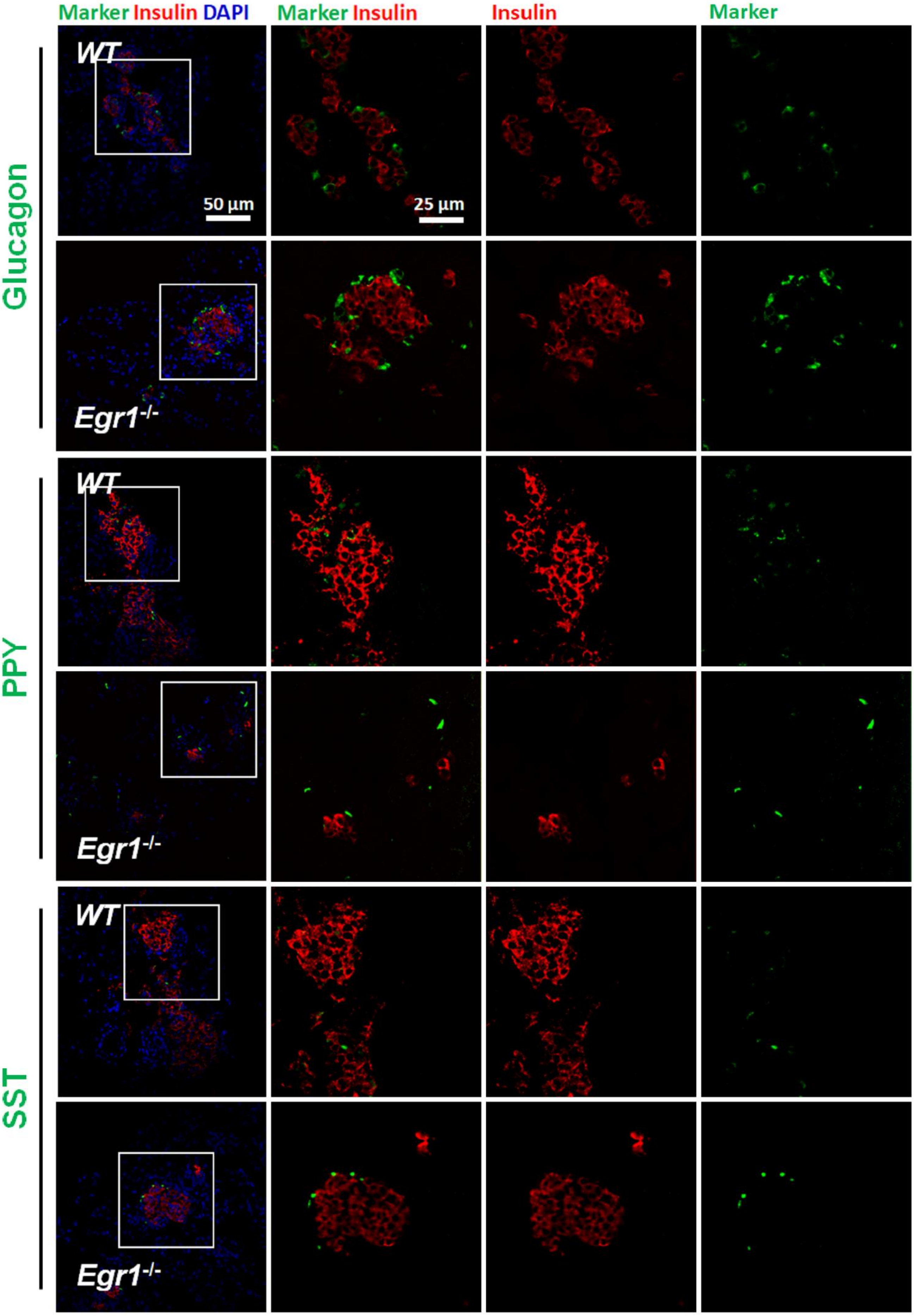

Marker Insulin DAPI
Marker Insulin
Insulin
Marker
Glucagon
PPY
SST
WT
Egr1-/-
50 µm
25 µm

Figure S4. Loss of β-cell identity in the islets of neonatal *Egr1*$^{-/-}$ mice. Confocal images of co-staining for the markers of β-cell (insulin) in *red* with α-cell (glucagon), PP-cell (pancreatic polypeptide, PPY), or δ-cell (somatostatin, SST) in *green* in the pancreas of postpartum day 1 (P1) neonatal mice. The enlarged images highlight the representative co-localization with 2× magnification from white squares in the overlay images.

Figure S5

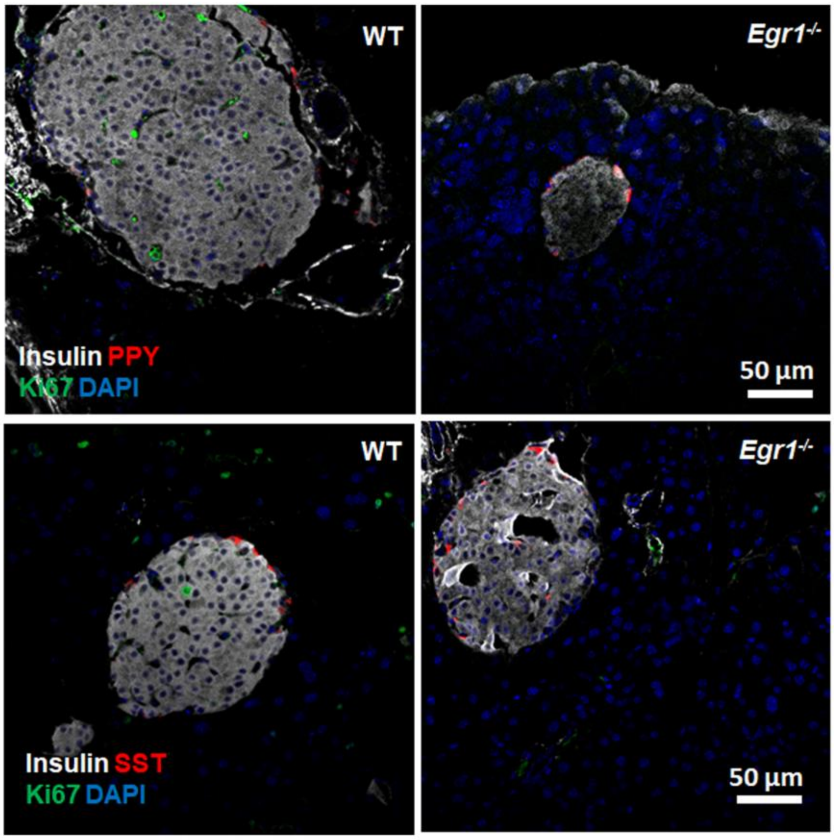

Figure S5. Ki67-positive PP- and δ-cells were not observed in HF-fed *Egr1*$^{-/-}$ mice. Immunofluorescence staining for Ki67 (*green*) with PPY or SST (*red*) and insulin (*white*) in the pancreas of 5-month-old male mice fed a HF diet for 3 months. Scale bar: 50 µm.

Figure S6

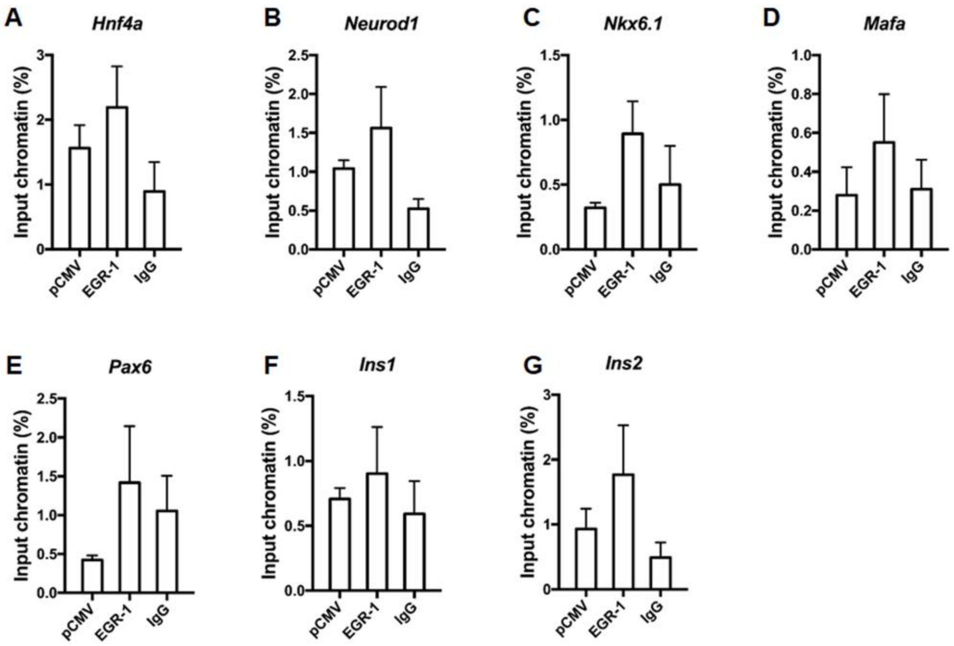

Figure S6. Chromatin immunoprecipitation assay in EGR-1 overexpressed and control (pCMV) MIN6 cells. Sequences containing the EGR-1 binding sites in potential target genes were amplified by real-time polymerase chain reaction. *n*=3 in each group.

Figure S7

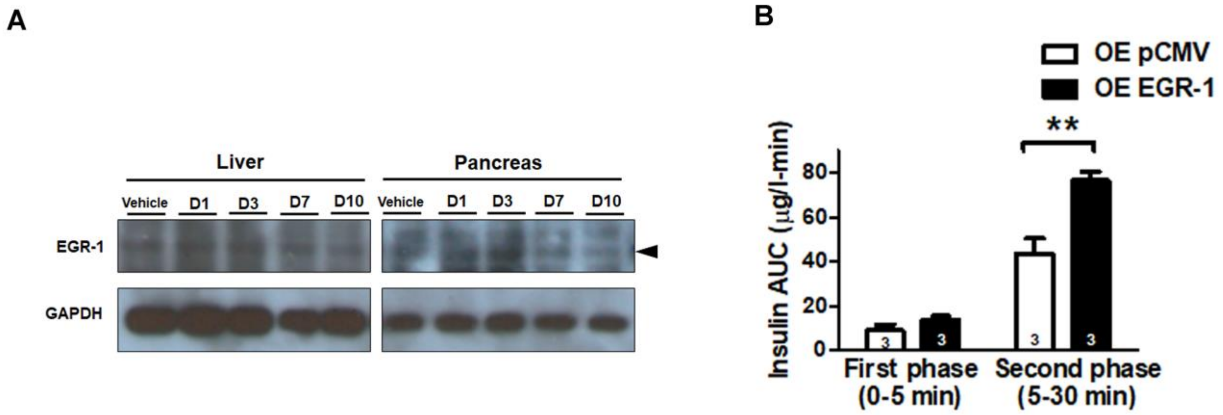

Figure S7. Overexpression of EGR-1 in mice. *A*: Immunoblot analysis on EGR-1 protein in WT mice received a single dose of EGR-1 plasmid (10 μg pCMV-EGR-1 plasmid/1.6 ml saline/mouse). The animals were euthanized at days 0, 1, 3, 7, or 10 after plasmid administration. Liver and pancreas tissues were analyzed for EGR-1 protein. *B*: *In vivo* glucose-stimulated insulin secretion (GSIS) in HF-fed WT mice received EGR-1 plasmid or empty vector pCMV (35 μg plasmid/1.6 ml saline/mouse) after oral challenge of glucose (4 g/kg body weight). Acute *in vivo* insulin secretion assay was performed at day 3 after plasmid administration. $^{**}P<0.01$.

Figure S8

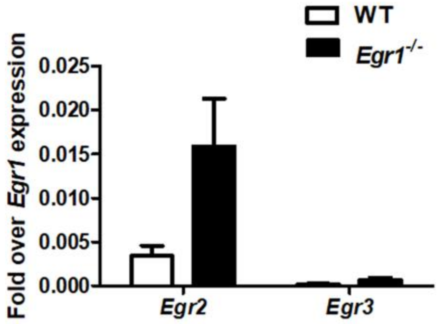

Figure S8. mRNA levels of EGR-2 and EGR-3 in the isolated islets of HF-fed *Egr1*$^{-/-}$ (*n*=9) and WT (*n*=5) mice. mRNA levels are expressed relative to the average expression of EGR-1 in WT mice.

Table S1. Sequences of primers used for real-time PCR.

Gene		Sequence	Amplicon
Egr1	Forward	GAACAACCCTATGAGCACCTGAC	101 bp
	Reverse	CGAGTCGTTTGGCTGGGATA	
Ins1	Forward	TATAAAGCTGGTGGGCATCC	186 bp
	Reverse	GGGACCACAAAGATGCTGTT	
Ins2	Forward	TTTGTCAAGCAGCACCTTTG	316 bp
	Reverse	GGTCTGAAGGTCACCTGCTC	
Hnf1b	Forward	CCCAGCAATCTCAGAACCTC	118 bp
	Reverse	AGGCTGCTAGCCACACTGTT	
Hnf1a	Forward	ACCACTGCATCCCTCCTATCA	129 bp
	Reverse	ACCTCAGGCTTGTGGCTGTAT	
Hnf3b	Forward	TCCGACTGGAGCAGCTACTAC	366 bp
	Reverse	GCGCCCACATAGGATGACA	
Hnf4a	Forward	GTTTAGCCGACAATGTGTGG	114 bp
	Reverse	TCCCGCTCATTTTGGACAG	
Mafa	Forward	CATCCGACTGAAACAGAAGC	200 bp
	Reverse	CCGCCAACTTCTCGTATTT	
Pdx1	Forward	GGATGAAATCCACCAAAGCTC	79 bp
	Reverse	TTGTTTTCCTCGGGTTCCGC	
Nkx6.1	Forward	GGACCAGAGAGAGCACGC	212 bp
	Reverse	TTCGGGTCCAGAGGTTTG	
Gck	Forward	GCACACGTGGTGCTTTTGAG	66 bp
	Reverse	GCCTTCGGTCCCCAGAGT	
Mafb	Forward	CCACCTCTTGCTACGTGTGA	199 bp
	Reverse	ACTGTACAACGGAAGGGACTTG	
Pax6	Forward	GCACATGCAAACACACATGA	96 bp
	Reverse	ACTTGGACGGGAACTGACAC	
Pax4	Forward	TCCTGTGGCTTCCTCCTCATA	129 bp
	Reverse	GAGGCCTCTTATGGCCAGTTT	
Neurod1	Forward	AGGAATTCGCCCACGCAGAAG	244 bp
	Reverse	CTCCTCTGCATTCATGGCTTCAAG	
Neurog3	Forward	GAGTTGGCACTCAGCAAACA	193 bp
	Reverse	TCTGAGTCAGTGCCCAGATG	
Gcg	Forward	ACCTGGACTCCCGCCGTGCCCA	197 bp
	Reverse	TCGCCTTCCTCGGCCTTTCACCAGCC	
Arx	Forward	GTTACCGCTTGTCCTGAGC	232 bp
	Reverse	GGCTCCCAGAAGCCTCATTT	
Sst	Forward	AGGACCTGCGACTAGACTGA	161 bp
	Reverse	GAAACTGACGGAGTCTGGGG	

Ppy	Forward	TAGCTCAGCACACAGGATGG	203 bp
	Reverse	GCCTGGTCAGTGTGTTGATG	
Nkx2.2	Forward	ACAACCCCTACACTCGCTG	214 bp
	Reverse	TAGGTCTGCGCTTTGGAGAAG	
Glut2	Forward	GTCCAGAAAGCCCCAGATACC	94 bp
	Reverse	GTGACATCCTCAGTTCCTCTTAG	
Gck	Forward	GCACACGTGGTGCTTTTGAG	66 bp
	Reverse	GCCTTCGGTCCCCAGAGT	
Kcnj11	Forward	GCTGCATCTTCATGAAAACG	298 bp
	Reverse	TTGGAGTCGATGACGTGGTA	
Abcc8	Forward	GGAAGGACTCACCACCATC	247 bp
	Reverse	GAGACCATCAAGGCGTAGG	
Pcna	Forward	TAAAGAAGAGGAGGCGGTAA	175 bp
	Reverse	TAAGTGTCCCATGTCAGCAA	
Cdk1	Forward	GGACCTCAAGAAGTACCTGGAC	90 bp
	Reverse	CCCTGGAGGATTTGGTGTAAG	
Ccna2	Forward	AGCAATGTTTTTGGGAGAAC	156 bp
	Reverse	AGGGTATATCCAGTCTGTTG	
Ccnd1	Forward	GCTGCAAATGGAACTGCTTC	191 bp
	Reverse	AGGGTGGGTTGGAAATGAAC	
Mki67	Forward	GCAGGTTAGCACTGTTATGAAAAC	115 bp
	Reverse	GGGCCTTGGCTGTTTTACATT	
Cdk5r1	Forward	GCCCTTCCTGGTAGAGAGCTG	113 bp
	Reverse	GTGTGAAATAGTGTGGGTCGGC	
Egr2	Forward	TCAGTGGTTTTATGCACCAGC	197 bp
	Reverse	GAAGCTACTCGGATACGGGAG	
Egr3	Forward	GTAGCCCATTACAATCAGATGGC	58 bp
	Reverse	CGTTGGTCAGACCGATGTCC	
PDX1	Forward	ATGAAGTCTACCAAAGCTCACG	208 bp
	Reverse	TGATGTGTCTCTCGGTCAAGTT	
CCND1	Forward	GAAGATCGTCGCCACCTG	61 bp
	Reverse	GACCTCCTCCTCGCACTTCT	
INS	Forward	CCTTTGTGAACCAACACCTG	223 bp
	Reverse	CTGGTACAGCATTGTTCCAC	
GCK	Forward	CATCTCTGAGTGCATCTCCGACT	253 bp
	Reverse	CGTGGCCACCGTGTCATTC	
EGR1	Forward	GCAGCAGCAGCACCTTCAA	112 bp
	Reverse	GGTAACTGGTCTCCACCAGCAC	

Table S2. Sequences of primers used for ChIP-PCR.

Gene		Sequence	Amplicon	Predicted binding site[1]	Other gene[2]
Pdx1	F	TGGCCACTAGGTAGATTATCTGTG	155 bp	-1697	0
	R	TGCCTCAATGAGTCCATTGTTCAG			
Arx	F	GCGTGCCAGCTGCTAATC	150 bp	-8	0
	R	GTCTCTCTGCTCCACGTGCT			
Hnf4a	F	GGGAAGGGTGTTACACAATGA	173 bp	26487 (intron 1)	0
	R	AAGAGGCCTTCGAGGAGAAA			
Neurod1	F	GAACCACGTGACCTGCCTAT	105 bp	-263	0
	R	GTCCGCGGAGTCTCTAACTG			
Nkx6.1	F	GGGGACAGAGCACAAACG	177 bp	67 (exon 1)	0
	R	TCCTTTTCGATCCGGCTAGT			
Mafa	F	CGCCCTCATTTGCCTATC	173 bp	-478	0
	R	GAATCTGCCACTTGGTCTCG			
Pax6	F	GAACCTAAGGACAGGCTACGG	154 bp	-222	0
	R	CTCCCTGAGGTTCGGCTTA			
Ins1	F	AGCAGGGCTTCTTACCCATT	189 bp	-1121518	0
	R	GGGTATAGGGGCGAAAGACT			
Ins2	F	AAGCTGTGGCTACCCTACCA	171 bp	161 (exon 2)	0
	R	GCATCTGCTCCCTCTACCAG			

***F, forward; R, reverse.**

[1]Location of predicted EGR-1 binding region for primer design.

[2]Number of other genes between predicted EGR-1 binding region and target gene.
